# Hemodynamic Traveling Waves in Human Visual Cortex

**DOI:** 10.1371/journal.pcbi.1002435

**Published:** 2012-03-22

**Authors:** Kevin M. Aquino, Mark M. Schira, P. A. Robinson, Peter M. Drysdale, Michael Breakspear

**Affiliations:** 1 School of Physics, University of Sydney, New South Wales, Australia; 2 Neuroscience Research Australia, Randwick, New South Wales, Australia; 3 School of Psychology, University of New South Wales, Kensington, New South Wales, Australia; 4 Brain Dynamics Center, Sydney Medical School – Western, University of Sydney, New South Wales, Australia; 5 Queensland Institute of Medical Research, Herston, Queensland, Australia; 6 School of Psychiatry, University of New South Wales, Sydney, New South Wales, Australia; 7 The Black Dog Institute, Sydney, New South Wales, Australia; 8 The Royal Brisbane and Women's Hospital, Brisbane, Queensland, Australia; Medical Research Council, United Kingdom

## Abstract

Functional MRI (fMRI) experiments rely on precise characterization of the blood oxygen level dependent (BOLD) signal. As the spatial resolution of fMRI reaches the sub-millimeter range, the need for quantitative modelling of spatiotemporal properties of this hemodynamic signal has become pressing. Here, we find that a detailed physiologically-based model of spatiotemporal BOLD responses predicts traveling waves with velocities and spatial ranges in empirically observable ranges. Two measurable parameters, related to physiology, characterize these waves: wave velocity and damping rate. To test these predictions, high-resolution fMRI data are acquired from subjects viewing discrete visual stimuli. Predictions and experiment show strong agreement, in particular confirming BOLD waves propagating for at least 5–10 mm across the cortical surface at speeds of 2–12 mm s-1. These observations enable fundamentally new approaches to fMRI analysis, crucial for fMRI data acquired at high spatial resolution.

## Author Summary

Functional magnetic resonance imaging (fMRI) experiments have advanced our understanding of the structure and function of the human brain. Dynamic changes in the flow and concentration of oxygen in blood are observed experimentally in fMRI data via the blood oxygen level dependent (BOLD) signal. Since neuronal activity induces this hemodynamic response, the BOLD signal provides a noninvasive measure of neuronal activity. Understanding the mechanisms that drive this BOLD response is fundamental for accurately inferring the underlying neuronal activity. The goal of this study is to systematically predict spatiotemporal hemodynamics from a biophysical model, then test these in a high resolution fMRI study of the visual cortex. Using this theory, we predict and empirically confirm the existence of hemodynamic waves in cortex – a striking and novel finding.

## Introduction

Functional magnetic resonance imaging (fMRI) experiments have substantially advanced our understanding of the structure and function of the human brain [1]. Hemodynamic responses to neuronal activity are observed experimentally in fMRI data via the blood oxygenation dependent (BOLD) signal, which provides a noninvasive measure of neuronal activity. Understanding the mechanisms that drive this BOLD response, combined with detailed characterization of its spatial and temporal properties, is fundamental for accurately inferring the underlying neuronal activity [2]. Such an understanding has clear benefits for many areas of neuroscience, particularly those concerned with detailed functional mapping of the cortex [3], those using multivariate classifiers that implicitly incorporate the spatial distribution of BOLD [4], [5], and those that focus on understanding and modeling spatiotemporal cortical activity [6]–[10].

The temporal properties of the hemodynamic BOLD response have been well characterized by existing physiologically based models, such as the balloon model [11]–[14]. Although the spatial response of BOLD has been characterized experimentally via hemodynamic point spread functions [15]–[18], it is commonly agreed that the spatial and spatiotemporal properties are relatively poorly understood [19], [20].

Many studies work from the premise that the hemodynamic BOLD response is space-time separable, i.e. is the product of a temporal HRF and a simple Gaussian spatial kernel. The latter assumed as a simple ansatz or ascribed to diffusive effects, for example [21]. This approach raises the following concerns: (i) since the temporal dynamics of the HRF is the focus of most theoretical analyses, e.g. the balloon model, this precludes dynamics that couple space and time, dismissing whole classes of dynamics, such as waves; (ii) in practice, employing a static spatial filter then convolving with a temporal HRF on a voxel-wise basis neglects non-separable interactions between neighboring voxels; and (iii) calculating temporal correlations between voxels then assumes that the hemodynamic processes responsible for the signal occur on scales smaller than the resolution of the measurements. In summary, neglecting spatial effects such as voxel-voxel interactions and boundary conditions (e.g., blood outflow from one voxel must enter neighboring ones) ignores important phenomena and physical constraints that could be used to increase signal to noise ratios and to improve inferences of neural activity and its spatial structure. These constraints are becoming increasingly relevant, as advances in hardware and software improve the spatial resolution of fMRI by reducing voxel sizes.

While treating spatial hemodynamics as a Gaussian is a reasonable first approximation [15]–[20], [22], this requires spatiotemporal BOLD dynamics, such as spatially delayed activity to be attributed solely to the underlying neuronal activity, without hemodynamic effects from neighboring tissue, an assumption that may not be valid. In this limit, BOLD measurements would simply impose a spatial low-pass filter of neuronal activity [20]. Several studies have already presented results that challenge this assumption, most strikingly by demonstrating reliable classification of neuronal structures such as ocular dominance or orientation columns [4], [5] on scales significantly smaller than the resolution of the fMRI protocols used [4], [5], [19], [23], [24]. Although there are suggestions that the organization of orientation columns may have low spatial frequency components, hemodynamics may also contribute to this effect. Going in the opposite direction, as voxels decrease in size, they must eventually become smaller in linear extent than the hemodynamic response, and thus become highly interdependent. Recent studies [20], [25]–[27], have highlighted how BOLD responses involve active changes in cortical vasculature, and hence reflect their mechanical and other spatiotemporal response properties, with spatial scales that are at least partly distinct from the scales of the underlying neuronal activity [20].

Given the above points, the mapping between neuronal activity and the spatially extended BOLD response cannot be assumed to be a spatially local temporal convolution [20], but should rather be treated in a comprehensive framework that accounts for both spatial and temporal properties and their interactions. A recent theoretical approach [25] treats cortical vessels as pores penetrating the cortical tissue and draws on a rich framework of methods developed in geophysics [28] to derive a physiologically based approximation of the hemodynamics. This model is expressed in terms of a closed set of dynamical equations that reduces to the familiar balloon model [25] in the appropriate limit where spatial effects are averaged over each voxel. It analyzes the spatiotemporal hemodynamic response by modeling the coupled changes in blood flow, pressure, volume, and oxygenation levels that occur in response to neural activity.

The objective of the present work is to make and empirically test novel predictions of the model, focusing particularly on spatiotemporal dynamics. We first predict the quantitative spatiotemporal hemodynamic response function (stHRF) for physiologically plausible parameters. We find that the model predicts a local response and damped traveling waves, whose speed and range are potentially observable with current high resolution fMRI. Second, we acquire and characterize such high resolution fMRI data from subjects viewing a visual stimulus designed to excite spatially localized neuronal activity in primary visual cortex. We observe hemodynamic waves in these experimental data, whose characteristics confirm our theoretical predictions of wave ranges, damping rates, and speeds, and constrain the physiological parameters of the model.

## Results

### Theoretical prediction: Spatiotemporal hemodynamic response function

**Figure 1:**
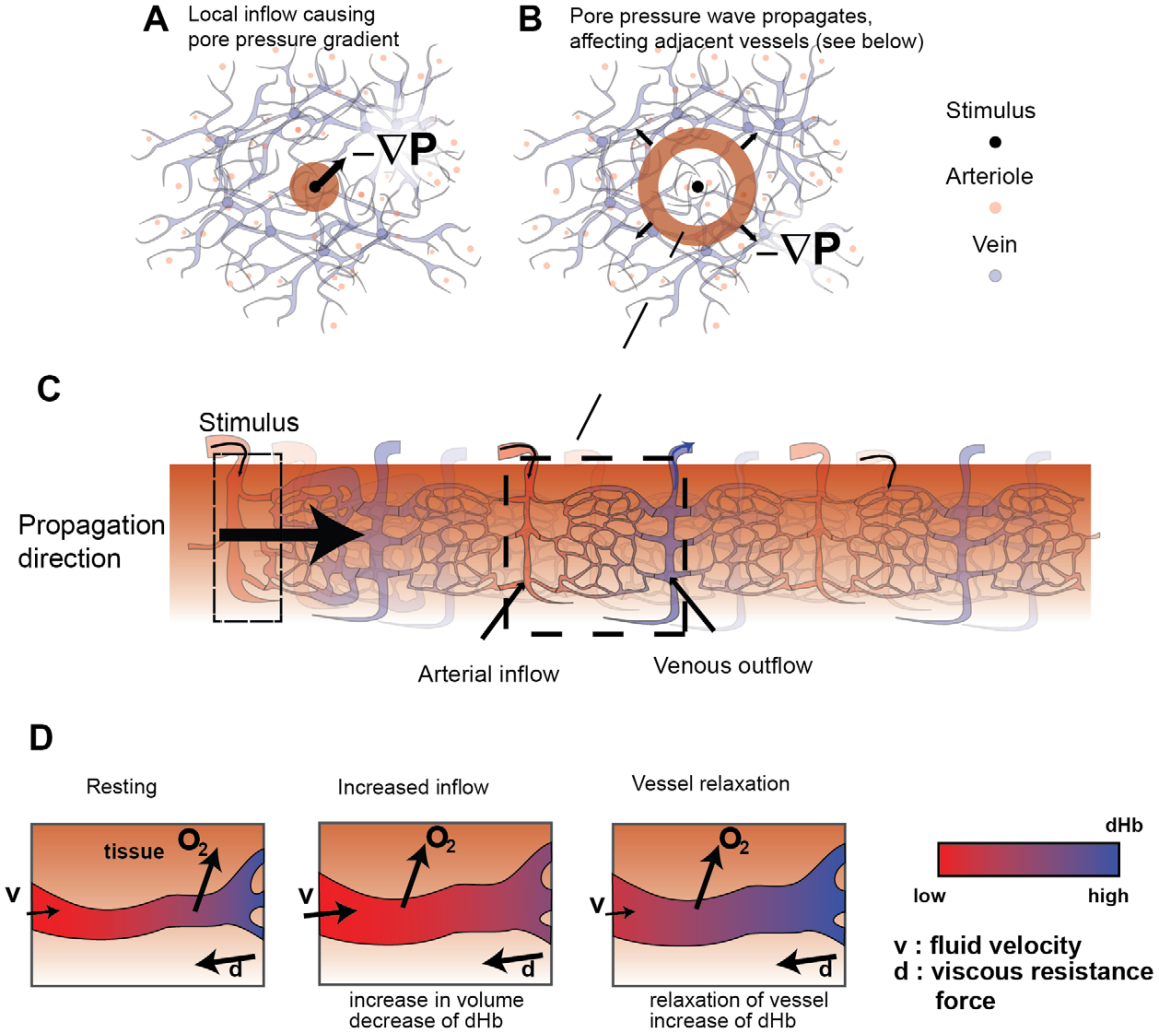
The spatiotemporal hemodynamic model. A: The hemodynamic response is driven by a localized spatiotemporal input, which represents neural activity and causes a change in arterial inflow of blood, which sets up a pressure gradient ∇P. B: This mass inflow deforms surrounding tissue and thus exerts pressure on nearby vessels. C: The rise in pressure causes further volume changes in adjacent vessels, which propagate outwards via interactions with successive regions of tissue. Damping of the response through blood viscosity and losses via outflow. D: The increase of local inflow increases oxygenated hemoglobin (oHb), reducing the amount of local deoxygenated hemoglobin (dHb). As oxygen is simultaneously passively extracted from blood, oHb is converted to dHb, causing a delayed rise of dHb during vessel relaxation.

Cortical hemodynamics and the resulting BOLD signal are modeled by incorporating the physiological properties of cortical vasculature into the theory of fluid flow through a porous elastic medium. The pores are the dense elastic cortical vasculature that penetrate the bulk cortical tissue [25]. In response to a rise in neural activity, local arterial inflow increases, deforming surrounding tissue and thus exerting outward pressure on neighboring tissue. The model predicts coupled dynamical changes in pressure, blood volume, and deoxyhemoglobin (dHb) content in the two-dimensional sheet comprising the cortex and its vascular layer (**Figure 1**). We refer the reader to the Methods for full details of the model.

Here we linearize this model and derive the stHRF, which is the BOLD response due to a spatially point-like, brief increase in neuronal activity. The main stages of this response are as follows: An increase in neuronal activity *z*(**r**,*t*) occurs, as a function of time *t* and position **r** on the cortical sheet. This causes relaxation of the smooth arterial muscles (mediated by astrocytes), inducing an increased influx of blood (**Figure 1A**) increasing local vessel volume and pressure. Blood then flows to regions of lower pressure, resulting in a redistribution of pressure through the medium (**Figure 1B**). These pressure changes induce further blood volume changes in adjacent cortical tissue. As a consequence of conservation of mass and momentum, this results in local changes of blood velocity in this adjacent tissue (**Figure 1C**). As these coupled changes propagate outwards, the viscous properties of blood lead to damping of their amplitude (**Figures 1C, D**). This is accompanied by outflows to draining veins, reducing vessel volume, and thus yielding further dissipation. Although these two sources of dissipation occur at small scales, they are reflected in the larger scale dynamics of the system.

Together, the above processes result in the propagation of pressure changes that travel beyond the ∼1 mm range of direct blood flow between adjacent arterioles and venules. In our theory, the time required for changes in directly coupled adjacent tissue to occur is comparable to the time it takes for the blood to transit the gray matter (∼1 s). Consequently, these considerations predict propagation speeds of order 1 mm/s.

During the change in vessel volume, local deoxyhemoglobin (dHb) content also changes (**Figure 1D**). The influx of arterial blood increases the content of oxygenated hemoglobin (oHb), hence reducing local dHb concentration. Further reductions occur by the removal of dHb by local outflow. As this process occurs, oxygen diffuses passively into the cortical tissue, converting oHb to dHb. Together, these processes result in an initial decrease of local dHb concentration followed by a delayed increase.

The BOLD signal thus reflects the net change of blood volume and dHb content. In the case of a spatiotemporally localized neural activation, the predicted BOLD response is given by the stHRF, expressed in Eqs. **1**–**5** of the Methods. The response to a more general stimulus is obtained by convolving the stimulus with the stHRF, as discussed in **Text S1**. Critically, the predicted stHRF (Eq. **5**) implies that the hemodynamic response contains a component that propagates as damped traveling waves over spatial scales potentially far greater than those of the neural signal that generated them. In other words, our model predicts that even if neuronal activity is restricted to a very small patch of cortex, it will cause changes in the BOLD signal that propagate for several millimeters over a few seconds.

**Figure 2:**
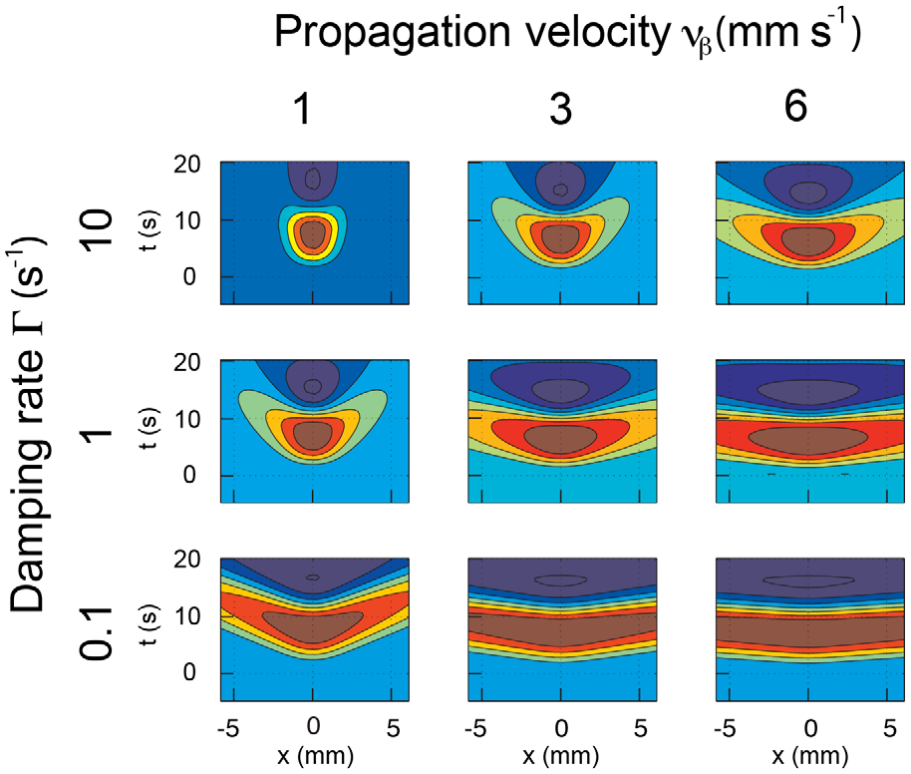
Predicted responses vs. *x* and *t* for a range of physiologically plausible values of of ν_β_ and Γ. Each column represents a particular *ν_β_* – as labeled at the top, while each row corresponds to a different Γ – as labeled at the left.

The precise quantitative properties of the predicted BOLD signal depend on several key physiological parameters that can be experimentally determined. Our analysis (**Figure 2**) indicates that the average vascular stiffness and the rate of damping due to blood viscosity and outflow at boundaries are the most critical parameters (see **Table S1 in Text S1** for a complete list of parameters). High stiffness results in a rapid return to equilibrium, thereby increasing the wave speed and range. Conversely high blood viscosity results in strong damping, thereby reducing the range of the waves. Figure 2 shows a range of predicted spatially extended BOLD responses spanning physiologically realistic ranges of these two parameters (**Text S1**). A combination of strong damping and low stiffness (**Figure 2**, top left panel) is predicted to lead to localized responses whose spatial extent is mostly confined to that of the neuronal signal. Although there would still be some weak signal propagation, it is unlikely this would be detectable given typical levels of measurement noise and voxel sizes. This parameter set corresponds to cases where the stHRF spatial scale is smaller than a typical voxel size, where the approach of treating voxels independently would be justified.

The opposite extreme of weak damping and high elasticity (**Figure 2**, bottom right) yields predicted responses that propagate rapidly and far across the cortical sheet. These parameters are unlikely to be relevant experimentally because such extensive waves would likely have already been reported. Between these two extremes there is a broad region of physiologically plausible values of these parameters (medium damping and/or vascular stiffness) for which traveling hemodynamic waves are predicted to have properties that are potentially detectable in current experiments but with ranges that would not likely have led to their detection to date.

### Experimental testing of theoretical predictions in human visual area V1

**Figure 3:**
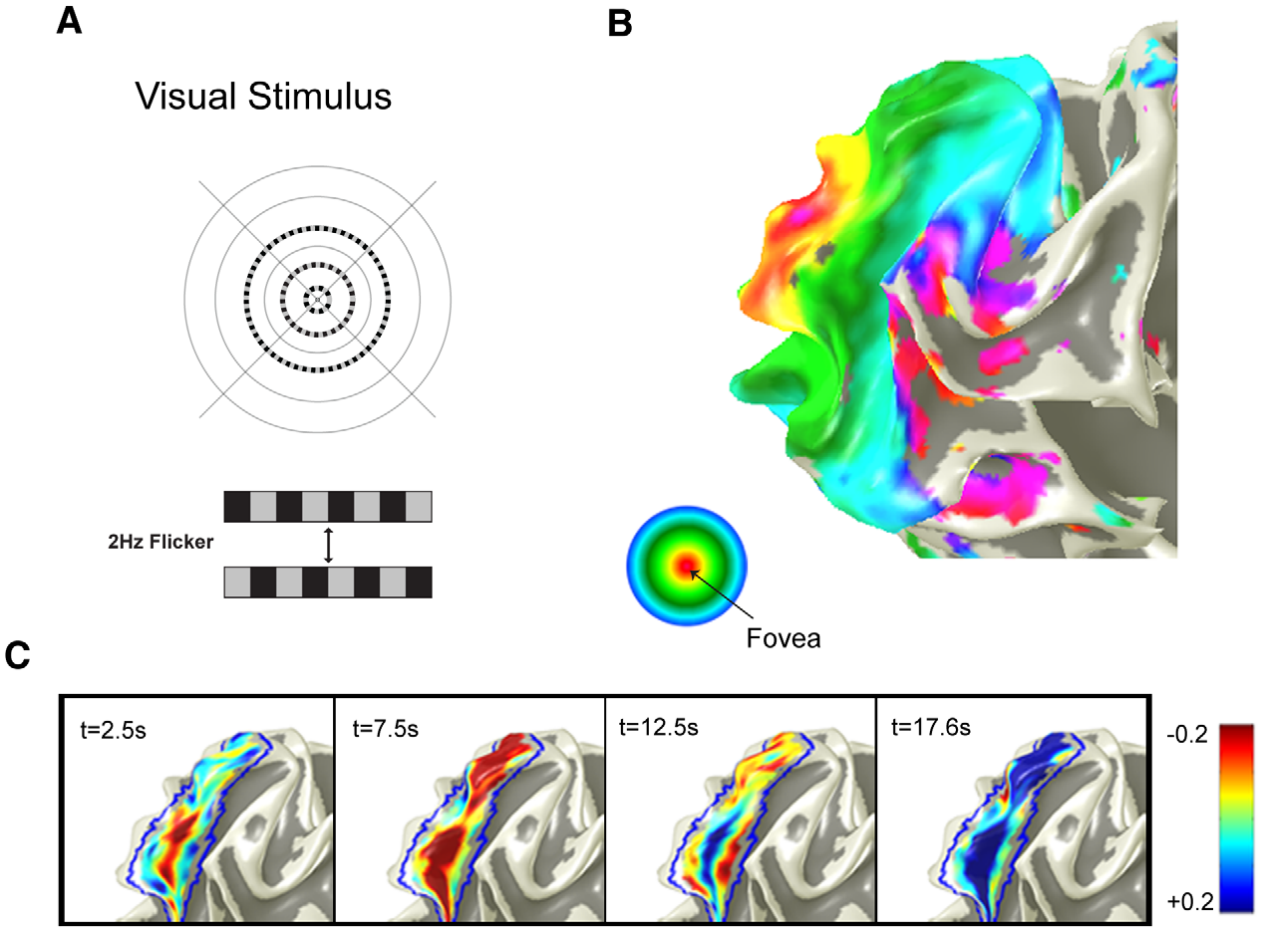
Experimental paradigm and evoked responses. **A:** The visual stimulus used in the experiment, where it is superposed on a gray background. The solid black circles and cross are always present, to aid fixation. The three dashed isoeccentric rings reverse 4 times per second, where the two patterns are shown at the bottom of this frame. **B:** Retinotopic mapping the allowed the locations of the 3 concentric rings to be independently identified, where the colors represent the measured eccentricity, colored from red at the fovea to blue at 6°, as shown in the circular inset, with magenta and gray at larger eccentricities [3]. **C:** Spatiotemporal snapshots showing the response to first ring at various times after stimulus onset. The colors represent percentage signal change, as labeled on the colorbar.

To test the predictions for the theory, high-resolution fMRI data were acquired in primary visual cortex, V1, from four healthy subjects. The well-known retinotopic mapping of the visual field to V1 allowed us to design simple visual stimuli that resulted in a spatiotemporally localized neural response [29]–[32]. Subjects viewed visual stimuli consisting of three dashed, time-varying (4 reversals of the light and dark dashes per second; i.e., a 2 Hz cycle) concentric rings at eccentricities of 0.6°, 1.6°, and 3° (**Figure 3A**). Subjects were instructed to focus at the center of the screen and perform a simple fixation task (see Methods).

These concentric visual stimuli resulted in strong BOLD modulations in early visual cortex, as seen in the example in Figure 3C. As a result of the retinotopic projection from visual field to early cortex (**Figure 3B**), these concentric rings are projected to lines on the cortical surface, one for each eccentricity. We find that the maximum BOLD modulation occurs on these lines, and that signal modulations weaken away from these peak responses. The three rings were presented at the same time. However, since the hemodynamic responses of the last two rings were not sufficiently separated on the cortex (in all subjects), we focus on the responses to the most inner ring, which was clearly separated (see Methods) from the responses of the other rings. The spatiotemporal evoked response is shown in a series of snapshots at different times *t* with respect to the stimulus onset (**Figure 3C**). Early responses (*t* = 2.5 s) are restricted to the central region (red) on these surface patches. As time progresses the BOLD signal near the center rises, and locations successively further from the center demonstrate increasingly delayed rises at *t* = 2.5–7.5 s. Finally, outward propagation of positive modulation in the periphery continues, while central responses decrease until they display the well known negative phase of the local post-stimulus undershoot at *t* = 12.5–17.6 s.

### BOLD waves on the cortical surface

**Figure 4:**
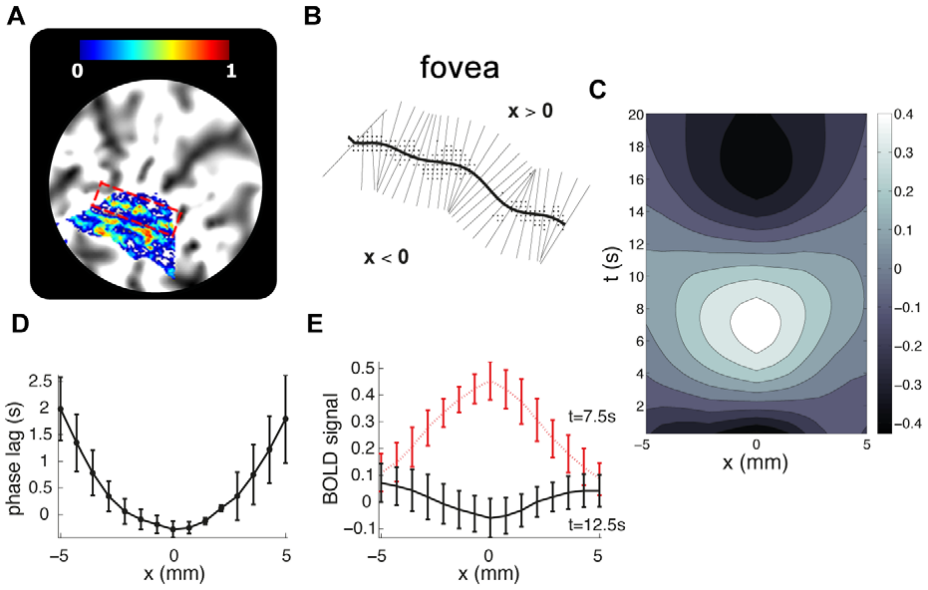
Traveling waves on the flattened cortical surface. **A:** Normalized Fourier power at the peak response frequency 0.1 Hz, calculated for voxels on the flattened surface, with the red rectangle outlining the response at 0.6° eccentricity (the line of high activation just below is at 1.6°). This power level reveals which voxels respond strongly to the experimental manipulation. **B:** Voxels that are coherent (Methods) with the stimulus (>0.4) provide an estimate of the approximately straight isoeccentricity curve on the cortex (the solid curve at *x = 0*). A polynomial curve is fitted to estimate the centerline, and perpendiculars are taken from this to find *x*. **C:** Averages of signals at all points with equal *x*, plotted vs. *x* and *t*, showing evidence of damped traveling waves in the form of sloping contours at left and right. The average percentage signal change ranges from −0.4 to 0.4 as indicated by the colorbar. **D:** Estimate of the 0.1 Hz signal delay from the stimulus onset vs. distance. **E:** Spatial cross-sections of the spatiotemporal response, relative to baseline, at *t* = 7.5 s (red), showing a clear rise in amplitude, and at *t* = 12.5 s (black), the central response has decreased and the surrounding signal remains above baseline.

The outward propagation (**Figure 3C**), discussed in the previous section, suggests the possibility of a traveling wave response, propagating normal to the centerline of the underlying neuronal response. We now focus on characterizing this response. Because stimulation of an isoeccentric curve in the visual field excites an approximately straight line of neurons in V1 [33], normal directions are clearly identifiable on a flattened cortex. We thus estimate the centerline of the primary isoeccentric response in a flattened representation of V1 (**Figures 4A,B**), and average the signal change over all points the same distance from this centerline (**Figure 4B**) (see Methods). Repeating this at various distances *x* and times *t* reveals the average spatiotemporal response (**Figure 4C**). Although the signal has been low-pass filtered in the *time* domain, and averaged along the direction parallel to the stimulus centerline, it is crucial to note that no *spatial* smoothing has been performed in the *x* direction.

The response has two characteristic spatial scales. Near the center (|*x*|<1 mm), a local response occurs, with a range similar to that of the expected neural response and whose time variation is similar to that predicted by the (purely temporal) balloon model. However, outside this region, the response propagates outward for several mm, with the peak response occurring steadily later as |*x*| increases (**Figure 4D**). At |*x*| = 5 mm the response is delayed, reaching its peak just as the central response |*x*|<1 mm reverses sign (**Figures 4D,E**). Furthermore, the amplitude of the propagating response decreases with |*x*| until it reaches the background level beyond |*x*| = 5 mm.

### Quantifying the spatiotemporal response

The above features of the BOLD signal modulations confirm the qualitative theoretical predictions of the spatiotemporal model. The theory also makes quantitative predictions of the waves' propagation speed, range, and damping rate. We next test these predictions and estimate the corresponding parameters through more detailed quantification of the empirical response.

**Figure 5:**
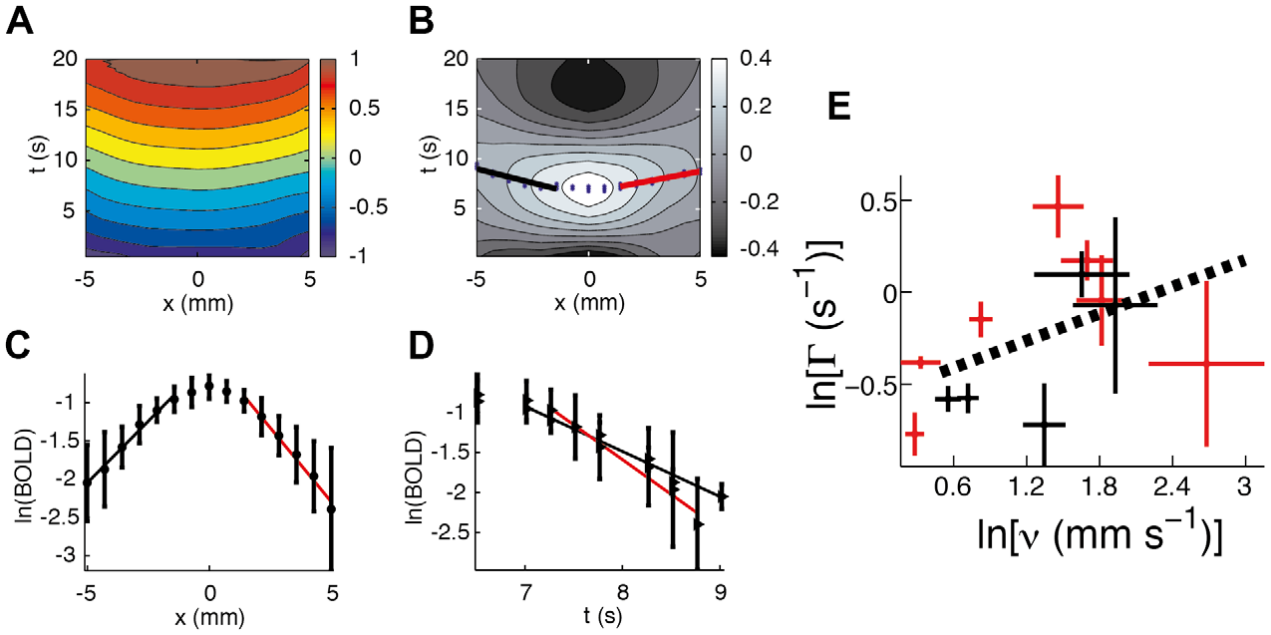
Quantifying the spatiotemporal response. **A** Calculation of the instantaneous phase reveal phase fronts of the response. These fronts are nearly stationary at the center and propagate with roughly constant velocity away from the center at |*x*|>1 mm. They are well described with straight line fits, as seen in panel **B:** toward the fovea (F) (red) and toward the periphery (P) (black). Along these slopes the BOLD signal decays exponentially in time **C:** with decay constants Γ_P_ = −0.56±0.04 s^−1^, Γ_F_ = −0.86±0.08 s^−1^, and **D:** as a function of perpendicular distance, with decay constants K_P_ = 0.32±0.02 mm^−1^, K_F_ = 0.38±0.02 mm^−1^. The error bars are 1 s.e.m, and errors of the parameter estimates are 1 s.d. **E:** Propagation velocity and damping rate for each of the subjects and their hemispheres. Red data points are the foveal values (F), while values toward the periphery (P) are shown in black. These points show a wide scatter, corresponding to individual differences, but there is a significant positive correlation. A straight line fit to this log-log plot yields an overall trendline that corresponds to a power law with exponent 0.24±0.09 (s.d).

To estimate the speed of wave propagation, phase fronts of the BOLD signal were estimated (see Methods). As the hemodynamic disturbance propagates, the spatially dependent time delay is evident (**Figures 3B** **,** **4D**). This delay also appears as a change in the phase of each frequency component of the response. Analysis of the instantaneous phase, at the frequency of maximum response (0.1 Hz), confirms propagation of phase fronts (**Figure 5A**). Points on the phase front that intersects the peak of the BOLD activity at *x* = 0 are overlaid on the spatiotemporal response in **Figure 5B**. This highlights the different behaviors of the nonpropagating local (|*x*|<1 mm) and propagating (|*x*|>1 mm) components of the BOLD response.

As depicted in **Figure 5**, our analysis shows that: (i) Near the centerline there is a localized response at approximately |*x*|<1 mm, corresponding to the spatial scale of the expected neural response [34], including the lateral spreading of thalamocortical projections from the lateral geniculate nucleus to V1 [35]. (note that all that is required to estimate the properties of the waves, in the following analysis, is that the central region, i.e. Δx, be small compared to the propagation distance of the waves so that we separate the propagating from the local component). (ii) Propagation occurs away from the center at a roughly constant speed (constant slope of the phase front) in both directions. Straight-line fits towards the fovea (F, red) and toward the periphery (P, black) yield propagation speeds *v*_F_ = 2.3±0.2 mm s^−1^ and *v*_P_ = 1.8±0.2 mm s^−1^ (s.d.), respectively for Subject 1 (**Figure 5B**). (iii) The signal is attenuated as it propagates, with fits to the log-linear plots (**Figure 5C**) yielding spatial damping constants *K*_P_ = 0.33±0.02 mm^−1^ and *K*_F_ = 0.39±0.02 mm^−1^ (s.d.). Characteristic ranges are the reciprocals of these constants; i.e., about 3 mm for the signal to decrease by a factor of *e*. Equivalently, the fits vs. *t* in (**Figure 5D**) imply temporal damping rates Γ_P_ = *K*_P_ *v*_P_ = 0.56±0.04 s^−1^ and Γ_F_ = *K*_F_ *v*_F_ = 0.86±0.08 s^−1^ (s.d.).

Data of sufficient quality to enable segmentation and primary response identification were obtained in seven of the eight available hemispheres. Data from the left hemisphere of one subject contained signal drop-out and artifact, most likely due to head movement, which prevented artifact-free surface-based reconstruction. Clear evidence of propagating waves in BOLD signal was observed in all seven of these usable data sets (**Figure S5**).

A total of 14 sets of wave responses were found, from which parameter estimates were able to be made for 12 (**Figure 5E**). Two cases of propagation toward the periphery in one subject showed interference from the second stimulus ring and were not used. These 12 responses yielded group averages *v* = 4±2 mm s^−1^, *K* = 0.28±0.03 mm^−1^, and Γ = 0.8±0.2 s^−1^ (s.e.m). The scatter plot (**Figure 5E**) shows a correlation (R^2^ = 0.22) between the temporal damping rate and the velocity. As discussed above, the ratio between these two quantities yield secondary estimates for spatial damping and are consistent with the substantially smaller relative uncertainty in the spatial damping constant. Furthermore, this shows that the spatial extent is relatively fixed regardless of the wave properties. This is consistent with the observation that the mean FWHM of the responses is clustered around 4.7±0.4 mm (s.e.m).

To recap, our biophysical theory shows that spatiotemporal hemodynamic responses obey a wave equation, whose key parameters are the wave velocity and a damping constant affecting decay in time and space. These parameters can be estimated from the empirical responses using simple regression analyses. We find that the parameter estimates here all lie within the a priori ranges estimated independently of the model (**Text S1**).

## Discussion

The increasing resolution of functional MRI and the development of analysis methods that depend on spatial patterns in these data underline the need for a systematic, quantitative approach to the spatial and temporal properties of the BOLD signal that is based on the properties of the underlying tissue and vasculature. Here we apply a recent physiologically based theory of hemodynamics in cortical tissue and vasculature to derive the linear spatiotemporal hemodynamic response function (stHRF), which is the response to a spatiotemporally localized stimulus of moderate amplitude. High resolution fMRI data are then used to test the predicted BOLD response to localized neuronal modulation in early visual cortex. Just two extra measurable parameters – *v*_β_ and Γ - suffice to characterize the spatial properties of the response.

The theory used is the first to make a mean-field approximation to cortical vasculature and goes beyond spatially point-like multicompartment models [12], [36], [37] as it allows calculation of spatiotemporal hemodynamic responses to general stimuli. It predicts, and the data demonstrate, that the hemodynamic response to a spatially highly localized neuronal drive exhibits traveling waves that propagate over a few mm of cortical tissue. Moreover, the velocity, damping and characteristic range of the observed waves are well within the range of theoretical predictions. These traveling waves have not been previously predicted or reported in human cortex. The central part of the response is non-propagating and has a temporal profile consistent with the standard balloon model [11]–[14], [38].

A further key implication of the spatial effects in our data is that fMRI voxels should only be treated independently if each voxel is larger than the stHRF scale. For interpreting fMRI acquired at sufficiently high spatial resolution the spatiotemporal properties of the stHRF must also be taken into account. Moreover, when voxels are small, we speculate that propagation of hemodynamic waves beyond their boundaries may underlie the observation that some experiments can be sensitive to structures at scales below the voxel size, including ocular dominance and orientation preference columns [4], [5], [24].

The combination of modeling and data allows us to estimate key physiological parameters of the model from observations of individual subjects. This lays the basis for replacing fMRI analysis procedures that rely on purely empirical analysis by ones that relate to the underlying physiology. We have shown how characterization of the spatiotemporal properties of fMRI data allows properties of the cortical tissue and vasculature to be inferred, hence accounting for differences between subjects and, potentially, brain regions. For example, relatively low blood viscosity and/or high tissue stiffness are predicted to lead to longer-range wave propagation. Specific experimental manipulations, such as the use of blood-thinning agents, could be employed to test the predicted changes in wave speed and spatial range. Similarly, the reduction in tissue elasticity that typically occurs with ageing [39], should be able to be probed noninvasively via its effects on wave velocity, and thereby taken into account when making inferences about neuronal activity in cohorts where age may be a confound. Likewise, regionally specific vascular properties have recently been highlighted as an important potential confound in studies of effective connectivity [40], [41], thereby underlining the need for a careful measurement and allowance for hemodynamic effects.

It is worth asking why hemodynamic waves have not been previously observed in fMRI. Some reasons are: (i) If voxel dimensions are large and sampled over a long time period, the hemodynamic response is not sufficiently resolved to detect propagating waves (ii) If the BOLD signal is spatially smoothed, then the spatiotemporal structure of the measured BOLD signal will be averaged out; (iii) Wave propagation is confined to occur within the cortical sheet and will be only be readily apparent in surface-based data reconstruction; (iv) Hemodynamic waves from a point source (e.g., a localized activation in a typical study) in two spatial dimensions decay more rapidly with distance than from the line source our experiment, where net decay can occur only in the direction perpendicular to the cortical locus of our one-dimensional stimulus. Despite these points, as high resolution protocols and surface-rendered data analysis techniques gain widespread use, the need for quantitative spatial analysis will likewise grow.

An important consequence of having hemodynamic traveling waves is that the spatial dynamics of BOLD are not independent of their temporal dynamics. The conventional factorization into spatial and temporal convolution operators is thus not valid in general. A greater understanding of the BOLD response, and brain mapping in general, would come from understanding the spatiotemporal hemodynamic response [20]. The present spatiotemporal HRF provides a solution to this problem, starting from a theory of spatiotemporal hemodynamics.

Several other issues arise from having hemodynamic traveling waves. (i) The existence of hemodynamic waves mean that spatiotemporal hemodynamics, induced by nearby sources, can interact in a nontrivial way, a property that occurs in the temporal domain, concerning the nonlinear interaction between temporally proximate responses [13], [38]. (ii) These findings cast further doubt on those measures of effective connectivity, such as Granger causality, unless they include a careful treatment of hemodynamic effects [40], [42], [43]. (iii) On the other hand, experimental designs could exploit the wave properties of hemodynamics by using stimuli that induce resonant properties of cortical tissue - akin to the temporal domain [14]- enhancing detection of the evoked signal.

Traveling waves of neuronal or glial origin have been described throughout the brain, including in visual cortex [43]–[46], raising the question of whether these waves might be responsible for the hemodynamic traveling waves seen in our data. However, several considerations argue against this: (i) The close match between the theoretically predicted values and the observed data strongly supports the conclusion that the waves in our data are of hemodynamic origin. (ii) Previous studies [45], [47], [48] that reported propagating neuronal waves in V1 of similar spatial extent to those seen here demonstrated that these waves are 1–2 orders of magnitude *faster* (approximately 200 mm s^−1^ in cats [47], 100–250 mm s^−1^ in primates [45] and 50–70 mm s^−1^ in rats [48]), and waves in cortical white matter travel even faster [43]. Likewise, although the spatial scales may be similar to those presently reported, the diffusion of nitrous oxide - which mediates the coupling between neuronal activity and vasodilation - occurs too rapidly to explain our results [21]. (iii) Another possible source of propagating signal of possible relevance are calcium waves traveling via astrocytes because these mediate the neuronal signal in vasodilation. However, these calcium waves travel at ∼10 µm s^−1^ [49], which is 2 orders of magnitude *slower* that the waves reported here.

Although hemodynamic waves have not been characterized, detected, or previously modeled, existing work has detailed some spatiotemporal properties of the BOLD response. Previous studies have demonstrated hemodynamic contributions to spatiotemporal BOLD response, including: the effect of draining veins [50], [51] which induces a latent BOLD signal due to these veins; effects across vascular layers [52], [53] that induce layer dependent delays of the BOLD response; and general effects of the vascular network [43] that cause delayed BOLD responses across extensive brain regions. Studies have also implemented ways to minimize such effects to improve spatial specificity of functional activations [43], [50]. The hemodynamic waves are different from the mentioned phenomena in that they exhibit propagation across the cortical surface. As the waves pass through they induce changes in arterioles, capillaries, and venules – not reliant on overall drainage by large veins. The possible interplay of these effects will be subject to future modeling and experimental work.

In summary, with advances in imaging technology and data analysis, intervoxel effects will become more pronounced, demanding spatiotemporal analyses based on the underlying brain structure and hemodynamics. By verifying a model that enables such analysis, the present paper opens the way to new fMRI probes of brain activity. These new possibilities include experiments using spatial deconvolution to discriminate between neural and hemodynamic contributions to the spatiotemporal BOLD response evoked by complex sensory stimuli. An important potential application would be to disentangle negative components of the BOLD response from surround inhibition in the visual cortex. Our analysis also affords novel insights and physiological information on neurovasculature, a subject of particular significance to ageing and vascular health. Finally, the combination of the present stHRF with spatially embedded neural field models [8] would allow a systematic and integrated computational framework for inferring dynamic activity in underlying neuronal populations from fMRI data.

## Methods

The methods used in this study are threefold: (i) Derivation of a theoretical spatiotemporal hemodynamic response function (stHRF) from a physiologically based model of cortical hemodynamics; (ii) Execution of a customized experimental protocol for the acquisition and preprocessing of high resolution fMRI data on the response to spatially localized visual stimuli; and (iii) Model-based analysis of these data and explicit comparison with the predicted stHRF, including inference of underlying cortical hemodynamic parameters.

### Theoretical prediction

The theoretical prediction of the stHRF is derived from a physiologically-based model for spatiotemporal hemodynamics [25]. This model treats brain tissue as a poroelastic medium, with interconnected pores representing the cortical vasculature. The governing equations are a set of nonlinear partial differential equations that connect blood flow velocity ***v***, mass density contributed by blood (i.e. the part of the total density contributed by blood as opposed to tissue) *ξ*, deoxygenated hemoglobin concentration *Q*, and blood pressure *P* due to an increase in arterial flow *F* caused by an increase neural activity *z*, as a function of time *t* and position on the cortex ***r***. Although we describe changes in blood volume throughout the text, we model changes in ξ - which is closely related to the fractional volume of blood in tissue: *ξ*/*ρ*_f_, where *ρ_f_* is the density of blood itself. These model equations were recently derived and explained in a separate paper [25]. We provide a synopsis here and apply this with appropriate boundary conditions (**Text S1**) to calculate the stHRF and derive a hemodynamic wave equation.

The dynamics of flow *F(* ***r*** *,t)* are modeled as a damped harmonic oscillator [13] driven by neural activity *z(* ***r*** *,t):*

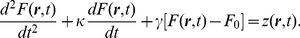
(1)The dynamics in Eq. **1** are parameterized by the signal decay rate κ, the flow-dependent elimination constant *γ* and the resting flow *F_0_*. The neural activity *z(* ***r*** *,t)* drives a distribution of arterial control sites (see **Figure S1**), as described further in **Text S1**.

The vascular response due to this increase in arterial flow is then constrained by physical laws, including conservation laws. Firstly, the conservation of blood mass is embodied by
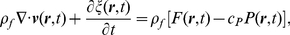
(2)where *c_P_* is a proportionality constant. This conservation law describes how the rate of change of local blood mass density ∂*ξ*/∂*t* is determined by the local divergence of the flow *ρ_f_*∇•(***v***), the source of mass due to the average inflow of blood *F*, and the average venous outflow of blood *c_P_P*. These latter source/sink terms are mean-field terms that describe the average spatiotemporal hemodynamic processes (For more details see the **Text S1** on the derivation of these terms).

The rate at which blood travels through the vasculature depends on the elastic response of cortical vessels. This process must conserve momentum, expressed as
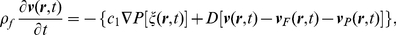
(3)where *P* is the average pore pressure, *D* parameterizes damping due to blood viscosity, and *c*_1_/*ρ_f_* is the constant of proportionality between pressure gradient and acceleration in the porous medium. This equation describes how forces are directed down pressure gradients, causing blood to accelerate toward regions of lower pressure. These velocity changes are resisted by blood viscosity leading to the resistive term *D*(***v*** **-** ***v*** *_F_* - ***v*** *_P_*) where ***v*** *_F_* and ***v*** *_P_* are the blood velocities at inflow and outflow, respectively (**Text S1**). The average pressure is related to the elastic properties of blood vessels by the constitutive equation, 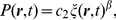
(4)where the elasticity of blood vessels is parameterized by the Grubb exponent 1/*β* (see **Table S1 in Text S1**) and a proportionality constant *c*_2_, as in previous empirical studies of cerebral blood flow [54].

As changes in local blood volume occur, oxygen diffuses into cortical tissue because of the increased partial pressure of oxygen. This process produces blood deoxyhemoglobin (dHb) - whose concentration is represented by *Q* - from oxygenated hemoglobin. The local concentration of hemoglobin in tissue is a fixed proportion *ψ* (in mmol kg^−1^), of local blood density in tissue, and is thus expressed as *ψξ*. Hence, the difference *ψξ* - *Q* is the amount of oxygenated hemoglobin. If *η* is the fractional rate at which oxygen passes from oxygenated hemoglobin to cortical tissue, the flow of dHb obeys a conservation equation, similar to Eq. **2** for the conservation of blood mass:
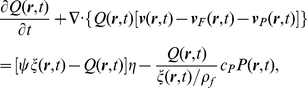
(5)where the difference term (*ψξ* - *Q*)*η* on the right hand side introduces the source of dHb, in which dHb is convereted to oHb at a rate *η*, and the term –*QPc_P_*/(*ξρ_f_*) represents the rate of reduction of dHb concentration due to blood outflow. This assumes that the blood is well mixed so the concentration leaving a vascular unit is *Q*/(*ξρ_f_*) at a rate of average venous blood outflow *c_P_P* (**Text S1**). As in Eq. **2**, the net outflow rate, here dHb ∇•(*Q* ***v***), is balanced by local changes in content, here ∂*Q*/∂*t*.

Finally, the measured BOLD signal *y* is predicted by a recent semi-empirical relation [55] between tissue blood volume content *ξ/ρ_f_* and dHb content Q to be
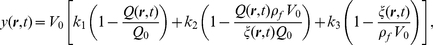
(6)where *V_0_* is the resting total volume, *Q_0_* is the resting fraction of dHb, and the constants *k_1_*, *k_2_*, and *k_3_* depend on the acquisition parameters, including field strength and echo time.

#### Calculation of the stHRF

Eq. **1**–**6** are a closed set of nonlinear partial differential equations which describe the hemodynamics in response to an arbitrary neural signal *z(* ***r*** *,t)*. To probe their fundamentals, we analyze their predicted spatiotemporal hemodynamic response function (stHRF) to a spatiotemporally localized stimulus, from which responses to general stimuli can be derived.

Provided the amplitude of neural activity is not too large, the model's response is approximately linear. Therefore, the hemodynamic variables henceforth represent linear perturbations from the steady state of hemodynamics. We thus approximate the hemodynamic response by linearizing the system **1**–**6** and arrive at the hemodynamic wave equation
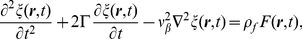
(7)where Γ is the damping parameter and *ν*_β_ is the propagation velocity. This equation predicts waves of *ξ* in response to an increase in local activity *F*. The damping parameter is given by 2Γ = *β*/*τ*+*D*/*ρ*_f_ and shows that dissipation of *ξ* waves is due to viscous damping, *D/ρ*_f_,and blood outflow *β*/*τ* at boundaries, which also dissipates wave amplitude. This equation represents the dominant factor that determines the traveling-wave part of the hemodynamic response (the rest of the equations are detailed in **Text S1**). Ranges for *ν*_β_ and Γ are of the order of mm s^−1^ and s^−1^, respectively (**Text S1**).

Fourier methods are next used to calculate a set of transfer functions, *T_Lz_*, which describe the ratio of the response of a hemodynamic quantity *L* (i.e., *Q*, *ξ*, *y*, or *F*) at a given spatial frequency (i.e., wave vector) ***k*** and temporal angular frequency *ω*, to the change in neural activity signal *z(* ***k*** *,ω)*. These transfer functions act as spatiotemporal filters and can be used to derive the BOLD frequency response to an arbitrary neural input. Hence, this includes the impulse response from a spatiotemporal neural input; i.e. the spatiotemporal HRF. This transfer function *T_yz_* maps from neural activity *z(* ***r*** *,t)*, to BOLD signal *y(* ***r*** *,t)*. The predicted experimental response *y(* ***r*** *,t)* is given by the inverse Fourier transform of the product of the transfer function in **Text S1: Eq. S17** and the Fourier transform of the neural activity *z*(***k*** *,ω*). To predict our experimental design we approximate the distribution of the evoked spatial neural activity as a Gaussian with a standard deviation of 1 mm in the *x* direction (**Text S1: Eq. S22**), to represent the spatial spread of the expected neural response [34]. The temporal neural response is simply the block design of the visual stimulus delivered in the experiment.

### General MRI procedures

By restricting the fMRI scans to the occipital pole, we achieved a resolution of 1.5×1.5×1.5 mm^3^ and 2 s TR echoplanar images (EPI). The stimulus onset was dephased by 250 ms per block for 8 blocks to further increase the effective time resolution. These EPI data were then coregistered to a high-resolution T1-weighted anatomical scan, acquired at 0.75×0.75×0.75 mm^3^, so that the spatiotemporal resolution was effectively resampled to 250 ms×(0.75×0.75×0.75) mm^3^. Furthermore, these mappings were restricted to the gray matter by segmenting the anatomical data into gray and white matter. Finally, functional retinotopic scans were used to map out the expected cortical positions in the visual cortex of each subject [3]. Data were acquired on a Philips 3 T Achieva Series MRI machine equipped with Quasar Dual gradient system and an eight-channel head coil.

### Subjects

Five healthy subjects (two female) ranging from 21 to 30 years participated in this study. The study protocols were approved by ethics boards of the University of New South Wales and Neuroscience Research Australia (formerly the Prince of Wales Medical Research Institute).

### Visual paradigm

Participants viewed visual stimuli via a mirror mounted on the head coil at a viewing distance of 1.5 m, resulting in a display spanning a diameter of 11° (or 5.5° in eccentricity). The visual paradigm was prepared with Presentation® software. Stimulus duration and fMRI pulse timing were logged with 0.1 ms accuracy. The stimulus consisted of 3 concentric rings simultaneously presented at 0.6°, 1.6°, and 3° eccentricity, presented in a block paradigm. (on for 8 s and off for 12.25 s). Each annulus was only 1 pixel wide (roughly 0.014° visual angle).

As known from retinotopic studies, isoeccentric lines in the visual field map to approximately straight lines in primary visual cortex. This stimulus was chosen to exploit this property, optimizing the identification of the primary response and secondary changes in BOLD signal in the orthogonal direction.

During the ‘on’ state, the annuli were divided into black and gray dashes that reversed roles 4 times per second (i.e., in a 2 Hz cycle). During the ‘off’ state, the annuli remained black (**Figure S2**). To improve visual fixation, a black fixation cross extended across the entire screen, 4 black circles were permanently present, and a pseudorandomly flickering fixation dot fluctuated between red, green, and blue [56]. Subjects reported that they were able to maintain alertness and attend to the fixation cross throughout data acquisition.

Note that off blocks were 12.25 s in duration, ensuring that the evoked response was effectively sampled at 250 ms. Each fMRI session consisted of 8 stimulus blocks, consisting of 80+1 fMRI volumes (the extra scan was due to the delayed onset) plus an additional 7 ‘off’ scans prior to the first block. Hence each session contained 88 fMRI volumes, a running time of 176 s, 14 such sessions were acquired from each subject.

To improve timing accuracy and synchronization we used a monitor refresh rate of 60 Hz for the visual display, and a TR of 2006 ms, rather than 2000 ms, to compensate for system delays. The remaining variability of the stimulus onset precision was logged and used for modeling the experimental design during data analysis.

### Functional data and preprocessing

To achieve high resolution, speed, and minimize distortions, we used a SENSE [57] accelerated echoplanar imaging (EPI) sequence. Great care was taken to minimize distortion, and each subject's data were carefully investigated to ensure distortion was minimal. Functional data were acquired in 29 1.5 mm slices for all but one subject, for whom there were 28 slices, with a 192×192 matrix, 230 mm field of view, and a SENSE factor of 2.3.

Functional data were motion corrected and slice scan-time corrected using SPM5 (SPM software package, http://www.fil.ion.ucl.ac.uk/spm/), then imported into the mrVista- Toolbox (http://white.stanford.edu/software/) for further processing and analysis. The fMRI data were transformed and analyzed in three different spaces: Firstly, the original planar space of the data acquisition, secondly, the 3 dimensional space defined by the high resolution T1 anatomy scans into which the data were aligned, transformed, and spatially up-sampled and finally for a flattened representation of the visual cortex, with maps of phase of the fMRI signals at the left and right occipital pole. Apart from the spatial up-sampling and mapping, no further preprocessing of the data in the spatial dimension was performed. The temporal time series of each voxel were low-pass filtered with a third order Butterworth filter below 0.1 Hz. Furthermore, although there were three concentric rings, we focus only on the 0.6° ring closest to the fovea when analyzing the spatiotemporal hemodynamic response as this was clearly spatially distinct whereas there was some overlap in responses to the furthest two rings (1.6°, and 3°).

### Distance measurements

Distance measurements were made along the cortical surface using meshes generated by the segmentation on each subject. A shortest path algorithm (in the VISTA software) was used to determine these distances on the surface.

### Isoeccentric averaging

When a stimulus excites a line of cortex, as in the present case, the hemodynamic response depends only on time and the perpendicular distance *x* from that line. To analyze these dependences, we estimated the location of the centerline of the primary response on the flattened surface, then measured the average BOLD signal at various distances orthogonal to this response as a function of time since stimulus onset. This was achieved in five steps (see **Text S1** for further details):

- Voxels with stimulus-related signal change were deemed to be those with signal fluctuations above a normalized temporal Fourier power of 40% relative to the total power at the peak stimulus response frequency bin of 0.1 Hz.
- Voxels within the primary visual cortex and at the approximate locations corresponding to the visual stimulus of 0.6° eccentricity were identified using a previously acquired retinotopic map as a spatial prior (**Figure 4A** **and Figure S3**).
- A polynomial was fitted to the spatially distributed voxels fulfilling these criteria (**Figure 4B**), and is an estimate of the centerline of the response. The order of this polynomial was estimated using the Aikake Information [58] criterion (see **Text S1 and Figure S4**).
- The distance *x* was found by constructing lines orthogonal to the centerline, extending 10 mm either side of it (**Figure 4B**) and measuring distances along them. Positive *x* was chosen to be toward the fovea.
- The spatiotemporal responses *y(* ***r*** *,t)* at all points with a given value of *x* were averaged at each time *t* to obtain *y(x,t)* (**Figure 4B**).

### Phase estimates with the Hilbert transform

As a hemodynamic disturbance travels, the BOLD signal phase depends on *x* and *t*. Phase fronts enable any wave propagation to be tracked at large |*x*|. To obtain phase estimates from the signal *y(x,t)*, we first constructed the analytic signal [59],
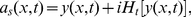
(8)where
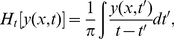
(9)is the temporal Hilbert transform [59]. The phase *ϕ*(x,t) is then given by
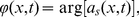
(10)where arg is the complex argument. Maps of this phase are shown in **Figure 5A** as well as in the Supplementary text for all the data sets. Constant-phase lines represent the phase fronts of the BOLD signal.

### Estimation of physiological parameters from data

The empirical estimates for the properties of the wave fronts were calculated from the phase fronts emerging from the peak of the BOLD signal at *x* = 0 (**Figure 5B**). From here, the two principal characteristics of the spatiotemporal response can be identified, the local response, close to *x = 0*, as a region of near-uniform phase spanning |*x*|<1 mm, and the propagating component heading away from these regions at |*x*|>1 mm (see **Figure 5B** and **Text S1**). This is consistent with the expected neural point spread function estimated from independent physiological data [34]. Straight-line fits to this propagating region, as shown in Figure 5B, yielded estimates of the wave velocity ν_β_.

The BOLD signal was measured at each point on the phase front as a function of time (**Figure 5C**) and space (**Figure 5D**). Transformation to logarithmic scales yielded approximately straight line plots, suggesting exponential decay of BOLD signal in space and time. Linear regression then yielded rate constants of temporal and spatial signal decay. Estimation of the standard error of these linear regressions provided error estimates for these parameters (see **Text S1**).

## Supporting Information

Figure S1 **The geometrical space for the hemodynamic response function.** The center of this corresponds to a small area that contains various flow control sides that all contribute to the injection of mass into the system.
(TIFF)

Figure S2 **The visual stimulus presented to subjects in the scanner.** The three rings;1,2, & 3 in the figure, were located at 0.6°, 1.6°, and 3° eccentricity respectively. These rings flickered back and forth with four reversals per second during the stimulus on phase. These rings were overlaid on a grey background and fixation grid consisting of rings and lines (in dark grey) that were always present. The sizes of the rings and the lines are exaggerated for clarity, and in the experimental design were 1 pixel wide. The intensities of the light and dark grays are also exaggerated on the spatial oscillation on each ring: 1, 2, & 3, and shown below on the 2 Hz flicker panel. During the off phase rings 1,2, & 3 were set at the luminosity of the fixation grid. Not shown here is the fixation task, which was a small square at the centre which pseudorandomly osciallated between red, green and blue. Lumonisty values were measured at: 1.1×10^6^ cd/m^2^ and 3×10^5^ cd/m^2^ for the light and dark grays respectively. The background gray was measured at 6×10^5^ cd/m^2^, and the dark stimulus fixation lines were measured at 1.5×10^5^ cd/m^2^. These lumonisity values were measured with a Minolta © CS-100 photometer under conditions matching those of the subjects in the scanner.
(TIFF)

Figure S3 **Thresholding the data in V1.** **A:** Frequency responses in all voxels in the occipital pole for each subject, denoted by SXH, (where X is subject number and H is the hemisphere), where the peak at is in red of all subjects. **B:** The spatial distribution of the Fourier peak frequency response, at 0.1 Hz, where the colors represent normalized power shown in the colorbar. These show the first ring (0.6° eccentricity and red band closest to the centre) in all subjects with the outer two rings, in most cases, combining. These panels are masked to only include V1 which is overlaid on the flattened occipital pole. **C:** The complementary eccentricity retinotopic map for each subject, with the colors representing the eccentricity in the visual field, indicated by the filled circle, where the centre represents the fovea the colors extend out radially to the periphery at 5.5° of eccentricity. Both **B** and **C** colormaps are overlaid on a curvature map of the cortex, where the intensities represent cortical curvature going from high in black to low in white.
(TIFF)

Figure S4 **Fitting the stimulus centerline for all subjects and hemispheres.** On the left of each panel is the Akaike Information Criteria (AIC) vs. the degree of the polynomial fitted to the thresholded voxels. On the right the polynomial fit at the minimal AIC with corresponding orthogonal lines. In Subject 1, right at high orders, orthogonal lines highly intersected so that the sampling data was effectively reduced significantly. This mean that the minimal AIC was not optimal in this dataset, therefore the choice was made to choose the first significant drop in AIC from the previous order, *n = 3*.
(TIFF)

Figure S5 **Spatiotemporal responses and parameter estimates for each subject and hemisphere.** The procedure is that used to obtain Figure 5 of the main text, as described there. **A:** The instantaneous phase. **B:** Spatiotemporal response, with estimated wave fronts overlaid in black toward the periphery (*x*<0) and in red towards the fovea (*x*>0). **C:** Amplitude vs. *x*. **D:** amplitude vs. *t*. The peripheral estimates for S3 left and right hemispheres contained few data points due to interference between the response from the ring at the next eccentricity, and are thus omitted from the subsequent analysis (see **Figure S3**). The errors quoted for parameter estimates throughout this manuscript are 1 standard deviation, estimated from linear regression fits to the data.
(TIFF)

Text S1 **We present the full linearized model (with the descriptions of the boundary conditions, and how the equations were linearized) and detail the complete model parameters and estimate their values.** We also provide extra details of the polynomial fitting routines shown in **Figures 4** **and S4**.
(PDF)
