# Supplementary figures and images for: Hemodynamic Traveling Waves in Human Visual Cortex
Source: PLoS Comput Biol. 2012 Mar 22;8(3):e1002435. doi: 10.1371/journal.pcbi.1002435 (PMC3310706; doi:10.1371/journal.pcbi.1002435)

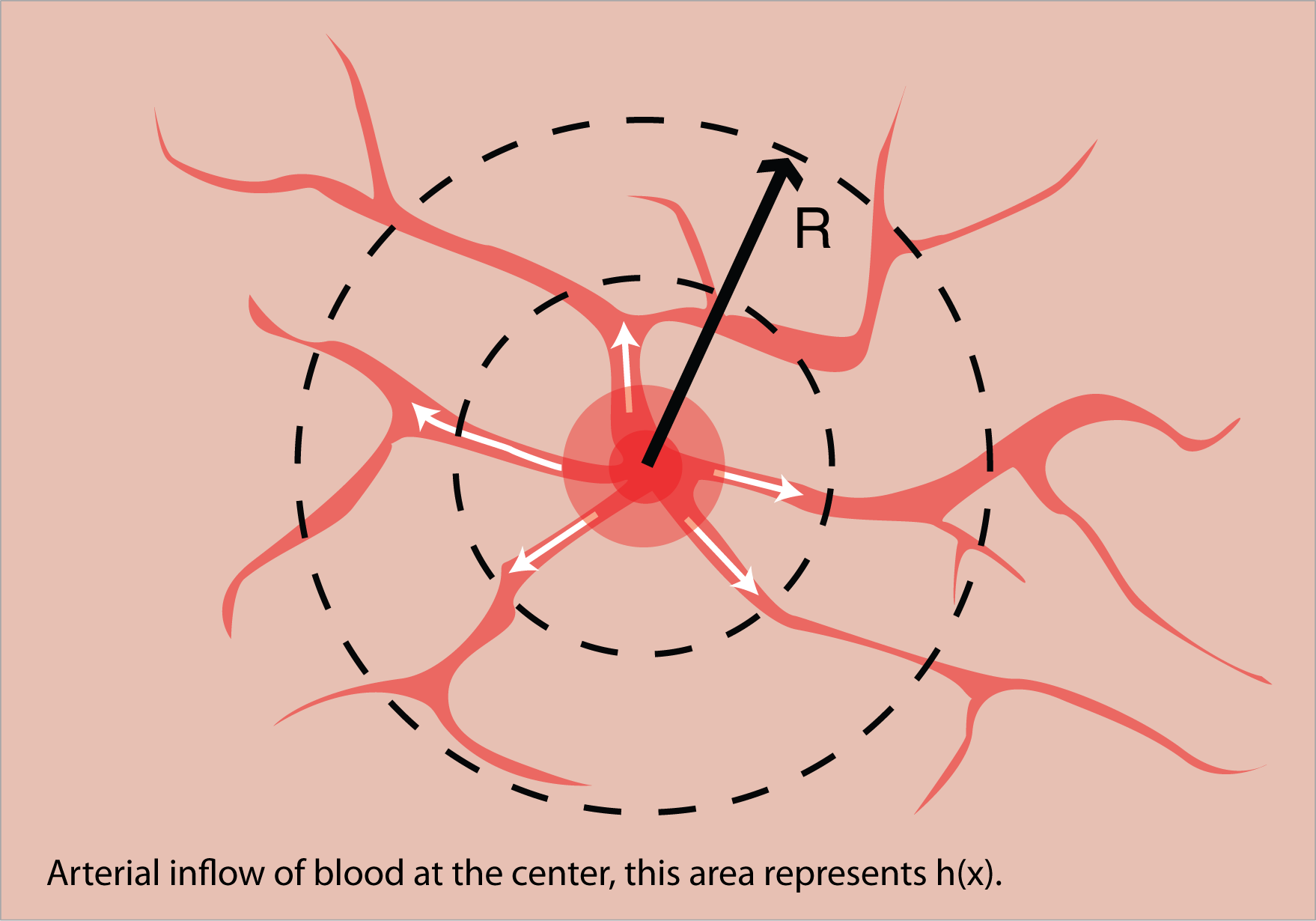

Supplement: Figure S1 — The geometrical space for the hemodynamic response function. The center of this corresponds to a small area that contains various flow control sides that all contribute to the injection of mass into the system. (TIFF) [file pcbi.1002435.s001.tif]

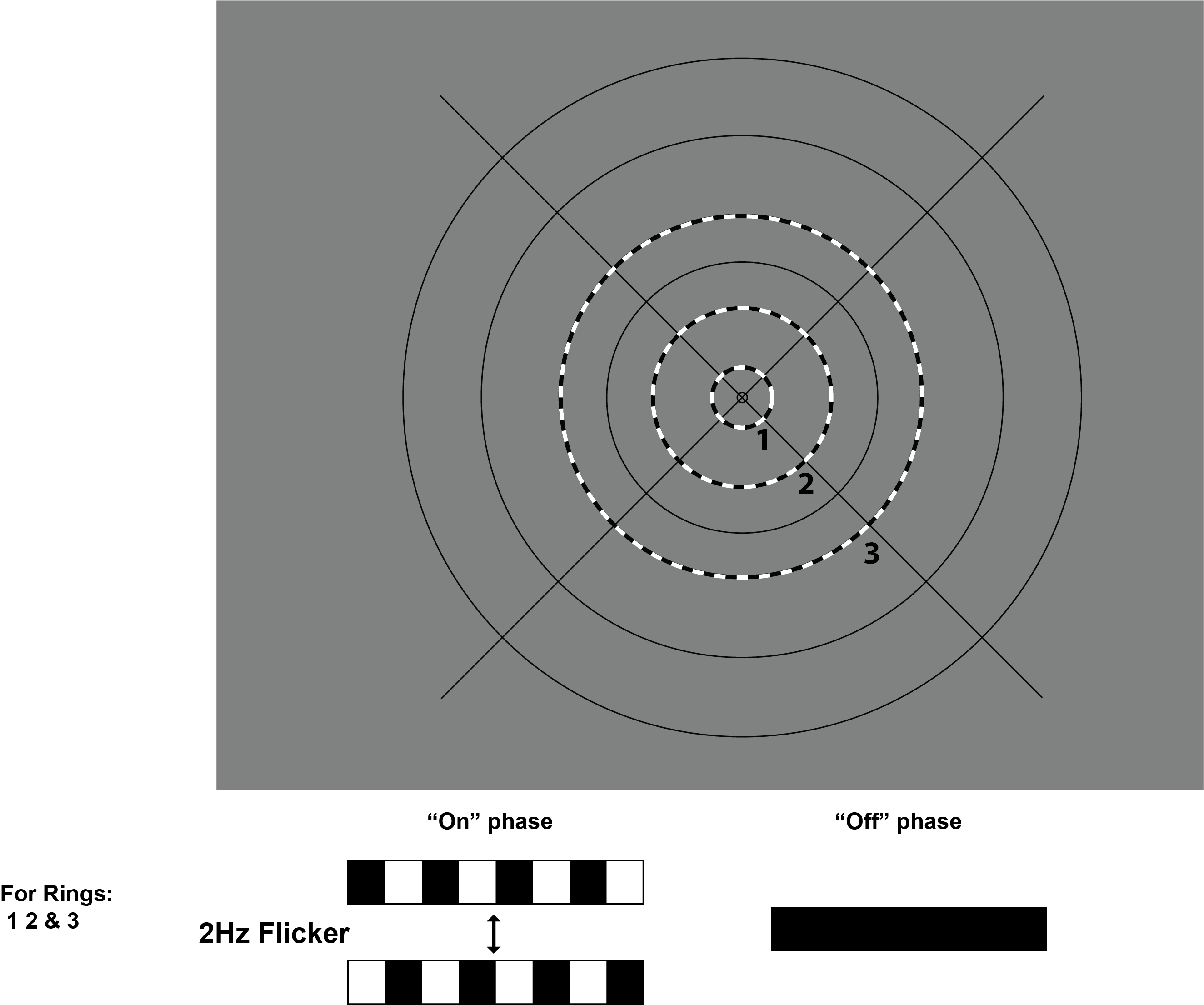

Supplement: Figure S2 — The visual stimulus presented to subjects in the scanner. The three rings;1,2, & 3 in the figure, were located at 0.6°, 1.6°, and 3° eccentricity respectively. These rings flickered back and forth with four reversals per second during the stimulus on phase. These rings were overlaid on a grey background and fixation grid consisting of rings and lines (in dark grey) that were always present. The sizes of the rings and the lines are exaggerated for clarity, and in the experimental design were 1 pixel wide. The intensities of the light and dark grays are also exaggerated on the spatial oscillation on each ring: 1, 2, & 3, and shown below on the 2 Hz flicker panel. During the off phase rings 1,2, & 3 were set at the luminosity of the fixation grid. Not shown here is the fixation task, which was a small square at the centre which pseudorandomly osciallated between red, green and blue. Lumonisty values were measured at: 1.1×10^6^ cd/m2 and 3×10^5^ cd/m2 for the light and dark grays respectively. The background gray was measured at 6×10^5^ cd/m2, and the dark stimulus fixation lines were measured at 1.5×10^5^ cd/m2. These lumonisity values were measured with a Minolta © CS-100 photometer under conditions matching those of the subjects in the scanner. (TIFF) [file pcbi.1002435.s002.tif]

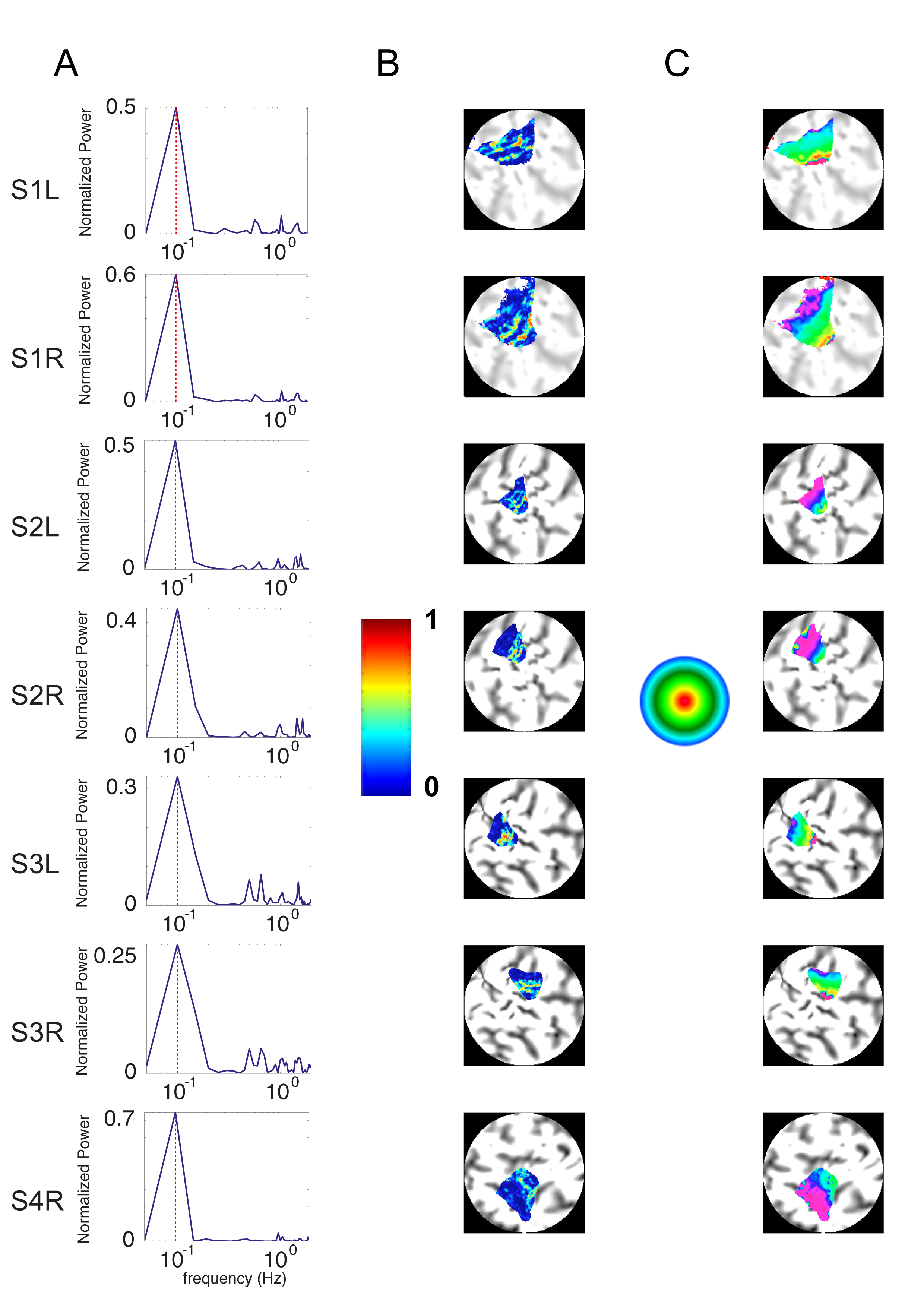

Supplement: Figure S3 — Thresholding the data in V1. A: Frequency responses in all voxels in the occipital pole for each subject, denoted by SXH, (where X is subject number and H is the hemisphere), where the peak at is in red of all subjects. B: The spatial distribution of the Fourier peak frequency response, at 0.1 Hz, where the colors represent normalized power shown in the colorbar. These show the first ring (0.6° eccentricity and red band closest to the centre) in all subjects with the outer two rings, in most cases, combining. These panels are masked to only include V1 which is overlaid on the flattened occipital pole. C: The complementary eccentricity retinotopic map for each subject, with the colors representing the eccentricity in the visual field, indicated by the filled circle, where the centre represents the fovea the colors extend out radially to the periphery at 5.5° of eccentricity. Both B and C colormaps are overlaid on a curvature map of the cortex, where the intensities represent cortical curvature going from high in black to low in white. (TIFF) [file pcbi.1002435.s003.tif]

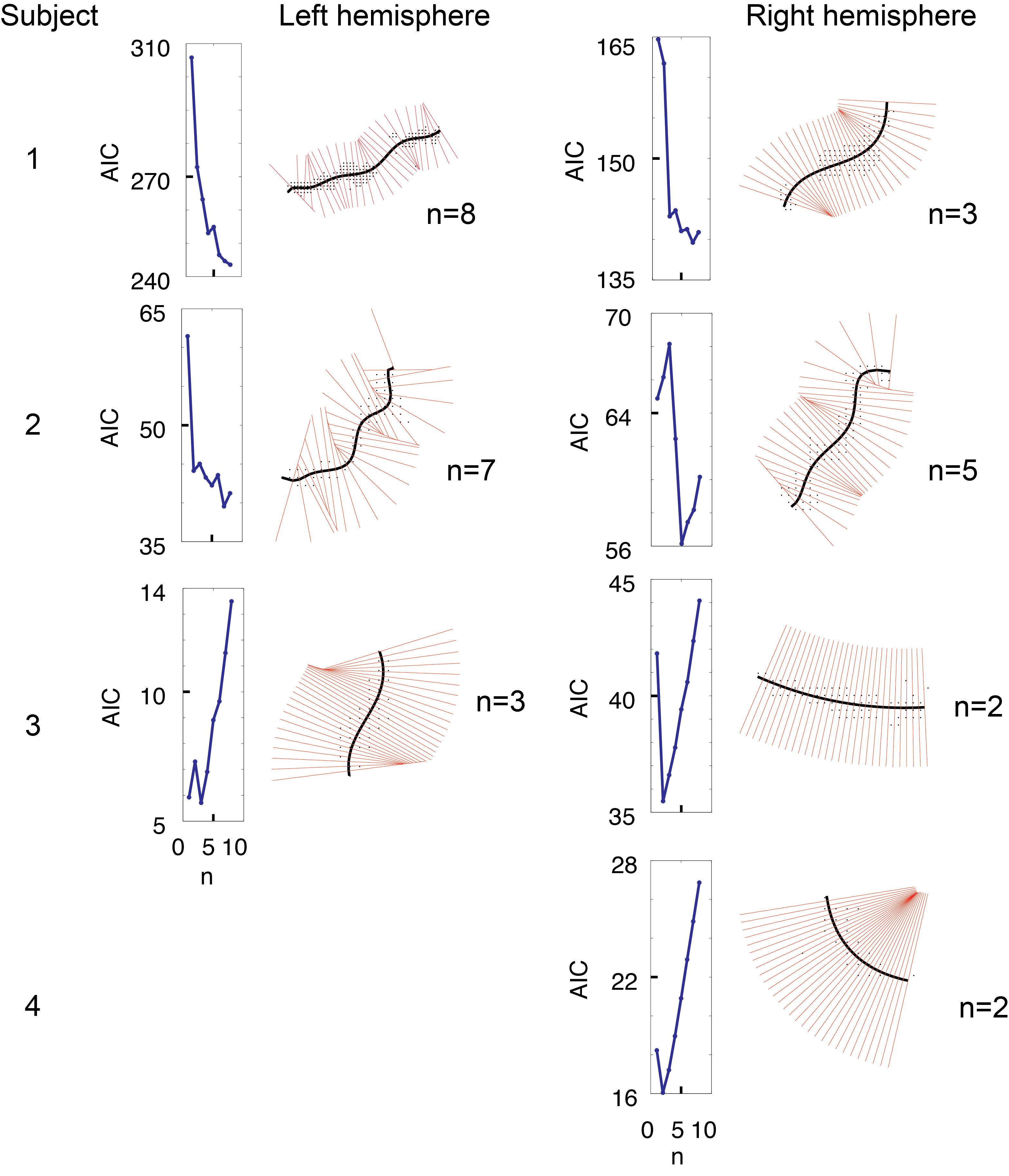

Supplement: Figure S4 — Fitting the stimulus centerline for all subjects and hemispheres. On the left of each panel is the Akaike Information Criteria (AIC) vs. the degree of the polynomial fitted to the thresholded voxels. On the right the polynomial fit at the minimal AIC with corresponding orthogonal lines. In Subject 1, right at high orders, orthogonal lines highly intersected so that the sampling data was effectively reduced significantly. This mean that the minimal AIC was not optimal in this dataset, therefore the choice was made to choose the first significant drop in AIC from the previous order, n = 3. (TIFF) [file pcbi.1002435.s004.tif]

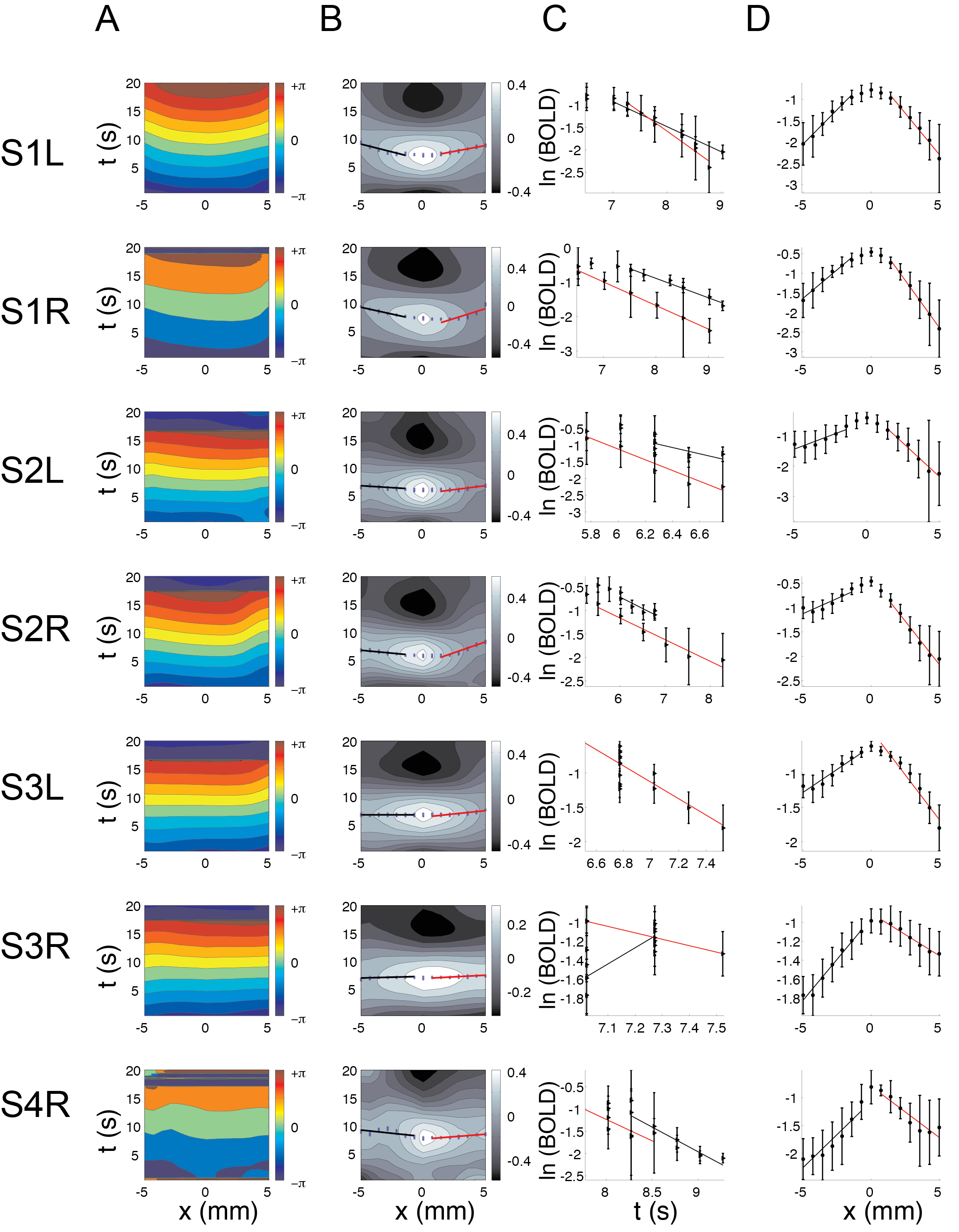

Supplement: Figure S5 — Spatiotemporal responses and parameter estimates for each subject and hemisphere. The procedure is that used to obtain Figure 5 of the main text, as described there. A: The instantaneous phase. B: Spatiotemporal response, with estimated wave fronts overlaid in black toward the periphery (x<0) and in red towards the fovea (x>0). C: Amplitude vs. x. D: amplitude vs. t. The peripheral estimates for S3 left and right hemispheres contained few data points due to interference between the response from the ring at the next eccentricity, and are thus omitted from the subsequent analysis (see Figure S3). The errors quoted for parameter estimates throughout this manuscript are 1 standard deviation, estimated from linear regression fits to the data. (TIFF) [file pcbi.1002435.s005.tif]
