# Supplementary material for: Hemodynamic Traveling Waves in Human Visual Cortex
Source: PLoS Comput Biol. 2012 Mar 22;8(3):e1002435. doi: 10.1371/journal.pcbi.1002435 (PMC3310706; doi:10.1371/journal.pcbi.1002435)
Supplement: Text S1 — We present the full linearized model (with the descriptions of the boundary conditions, and how the equations were linearized) and detail the complete model parameters and estimate their values. We also provide extra details of the polynomial fitting routines shown in Figures 4 and S4. (PDF) [file pcbi.1002435.s006.pdf]

Supplementary Material

Text S1

Spatiotemporal hemodynamics

The model equations described in [1] detail the general hemodynamic response. In the present study, the linear spatiotemporal hemodynamic response function (stHRF) is derived from this general model. Further mathematical analysis will be published in a subsequent paper. This document references equations from the Methods section of the main text by number, while equations from the supporting information are prefixed S.

The key steps in this derivation involve the definition of boundary conditions. These specify the inflow and outflow of blood mass, and thus appear as sources and sinks in the continuity equation relating the density of mass contributed by blood in tissue ξ to fluid velocity $\boldsymbol{v}$,

$$\rho_f \nabla \cdot \boldsymbol{v}(\boldsymbol{r},t) + \frac{\partial \xi(\boldsymbol{r},t)}{\partial t} = \rho_f \left(Source - Sink \right), \qquad \textbf{(S1)}$$

where ρ_f is the density of blood, and the source and sink on the right hand side are functions of time t and position $\boldsymbol{r}$ within the cortical tissue, and are in units of blood flow (s^{-1}). The source of blood mass is due to rise of neural activity, z, which modulates blood flow, $F(\boldsymbol{r},t)$, in a small region (See **Figure S1**). The sink of blood mass is due to the outflow of blood at draining veins and the rate at which this occurs is proportional to the pore pressure, $c_P P(\boldsymbol{r},t)$, where c_P is the constant of proportionality between pressure and blood outflow rate, listed in **Table S1**. Together these two conditions lead to the model equation given in Eq. **2** in the Methods.

The source and sink each correspond to boundary conditions on the fluid velocity terms that enter the mass and momentum conservation equations. By considering the flux of blood velocity to the mass inflow entering the system, the condition for the inflow fluid velocity $\boldsymbol{v}_F$ can be written as

$$\nabla \cdot \boldsymbol{v}_F(\boldsymbol{r},t) = F(\boldsymbol{r},t). \qquad \textbf{(S2)}$$

This argument is similar for the outflow condition, leading to

$$\nabla \cdot \boldsymbol{v}_P(\boldsymbol{r},t) = -c_P P(\boldsymbol{r},t), \tag{S3}$$

where $\boldsymbol{v}_P$ is the velocity at the outflow. At the microscopic level, mass inflow/outflows occur at discrete sources and sinks, respectively. In the present model the inflows and outflows are averaged over the mesoscopic scale (~0.5 mm) so that they are approximated as spatially continuous on the cortical sheet.

The constraints Eq. **S2** and Eq. **S3** on the fluid velocity then yield the momentum conservation Eq. **3** in the main text, with the corresponding Eq. **5** for the conservation of deoxygenated hemoglobin (dHb). Another boundary condition that needs to be specified is the rate at which the concentration of dHb leaves due to blood outflow. The form that is adopted is similar to balloon models, in that it argues that the outflowing blood is well mixed. This implies the concentration of dHb leaving the system is

$$Q(\boldsymbol{r},t)\frac{c_P P(\boldsymbol{r},t)}{\xi(\boldsymbol{r},t)/\rho_f}, \tag{S4}$$

which is the final term in Eq. **5** of the main text.

Model Linearization

As long as the neural activity signal is sufficiently small, the hemodynamic response can be estimated through linear analysis. Under this assumption one can analyze linear perturbations from the steady state. This is achieved mathematically by writing each variable θ [i.e., either $F,Q,P,\boldsymbol{v},z,$ or ξ] as the sum of its steady value θ_0 and its linear perturbation θ_1, i.e.

$$\theta(\boldsymbol{r},t) = \theta_0 + \theta_1(\boldsymbol{r},t). \tag{S5}$$

In this system, the steady state is determined by setting all spatial and temporal derivatives to zero and solving the ensuing algebraic equations. A further assumption is that the steady state on average is spatially uniform and that the mean blood fluid velocity averages to 0.

With these assumptions, the system dynamics can now be represented by four evolution equations. Firstly the wave equation for ξ [Eq. **7** of the main text] is

$$\frac{\partial^2 \xi(\boldsymbol{r},t)}{\partial t^2} + 2\Gamma \frac{\partial \xi(\boldsymbol{r},t)}{\partial t} - \upsilon_\beta^2 \nabla^2 \xi(\boldsymbol{r},t) = \rho_f F(\boldsymbol{r},t), \qquad \textbf{(S6)}$$

where $2\Gamma = \beta/\tau + D/\rho_f$ (Note that the superscript is omitted and from this point all quantities are linear perturbations from steady state). The linearized equation for dHb concentration is

$$\frac{\partial Q(\boldsymbol{r},t)}{\partial t} = Q_0 \frac{\partial \xi(\boldsymbol{r},t)}{\partial t} + \left[\psi \xi(\boldsymbol{r},t) - Q(\boldsymbol{r},t)\right]\eta - \frac{1}{\tau}\left[Q(\boldsymbol{r},t) + \frac{(\beta - 1)}{\xi_0}\xi(\boldsymbol{r},t)\right]. \qquad \textbf{(S7)}$$

The linear equation that links neural activity to blood flow is,

$$\frac{d^2 F(\boldsymbol{r},t)}{dt^2} + \kappa \frac{dF(\boldsymbol{r},t)}{dt} + \gamma F(\boldsymbol{r},t) = z(\boldsymbol{r},t)\,, \qquad \textbf{(S8)}$$

and the BOLD signal equation is

$$y(\boldsymbol{r},t) = \frac{1}{\rho_f}\left[(k_1 + k_2)\frac{\xi(\boldsymbol{r},t)}{\xi_0} - (k_2 - k_3)\frac{Q(\boldsymbol{r},t)}{Q_0}\right]. \qquad \textbf{(S9)}$$

Further details will be discussed in a companion paper that elaborates on the derivation of this wave equation and provides an extensive parameter exploration of the model.

Fourier analysis of linear perturbations

This linear response is analyzed in terms of its frequency content to derive complex spatiotemporal transfer functions $T_{AB}(\mathbf{k},\omega)$ that give the response of one variable A to changes in another B at the same spatial frequency (i.e. wave vector) $\mathbf{k}$ and temporal angular frequency ω via,

$$T_{AB}(\mathbf{k},\omega)=A(\mathbf{k},\omega)/B(\mathbf{k},\omega). \qquad \textbf{(S10)}$$

The frequency response to an arbitrary stimulus can be derived by Fourier means via these transfer functions. . The convention for the Fourier transform $f(\boldsymbol{k},\omega)$ with spatial frequency $\mathbf{k}$ and temporal angular frequency ω of a signal $f(\boldsymbol{r},t)$ is defined as

$$f(\boldsymbol{k},\omega) = \int d^2\boldsymbol{r} \int dt\, f(\boldsymbol{r},\boldsymbol{t}) e^{i(\omega t - \boldsymbol{k}\cdot\boldsymbol{r})}, \qquad \textbf{(S11)}$$

with its inverse being

$$f(\boldsymbol{r},t) = \int \frac{d^2\boldsymbol{k}}{(2\pi)^2} \int \frac{d\omega}{2\pi} f(\boldsymbol{k},\omega) e^{-i(\omega t - \boldsymbol{k}\cdot \boldsymbol{r})}. \quad \textbf{(S12)}$$

The following transfer functions were calculated for all the dynamical Eqs. **S6-S9**, yielding,

$$\mathrm{T_{Fz}}(\boldsymbol{k},\omega) = \frac{1}{-\left(\omega + \frac{1}{2} i\kappa\right)^2 + \omega_f^2}, \quad \textbf{(S13)}$$

$$\mathrm{T_{\xi F}}(\boldsymbol{k},\omega) = \frac{-i\omega\rho_f}{k^2 v_\beta^2 - \omega^2 - 2i\Gamma\omega}, \quad \textbf{(S14)}$$

$$\mathrm{T_{Q\xi}}(\boldsymbol{k},\omega) = \frac{Q_0}{\xi_0} \frac{1}{-i\omega + \eta + \tau^{-1}} \left[-V_0 i\omega - \tau^{-1}(\beta - 2) + \eta\right], \quad \textbf{(S15)}$$

These equations embody the physical stages seen in Fig. 1 of the main text. Eq. **S13** represents flow dynamics in response to a neural activity, Eq. **S14** represents the blood volume response to a rise in flow, and Eq. **S15** represents the response of dHb dynamics to a change in blood volume. Together these transfer functions for individual processes yield the overall transfer function via the BOLD signal Eq. **S9**:

$$\mathrm{T_{yz}}(\boldsymbol{k},\omega) = \frac{(k_2 - k_3)}{\rho_f} \left[1 - \frac{\xi_0}{Q_0}\left(\frac{k_1 + k_2}{k_2 - k_3}\right) \mathrm{T}_{Q\xi}(\boldsymbol{k},\omega)\right] \mathrm{T_{\xi z}}(\boldsymbol{k},\omega). \quad \textbf{(S16)}$$

Using **S13** –**S15**, the transfer function **S16** for the BOLD signal equation can be rewritten as

$$\mathrm{T_{yz}}(\boldsymbol{k},\omega) = \left[1 - \left(\frac{k_1 + k_2}{k_2 - k_3}\right) \frac{-V_0 i\omega - \tau^{-1}(\beta - 2) + \eta}{-i\omega + \eta + \tau^{-1}}\right] \frac{-i\omega(k_2 - k_3)}{k^2 v_\beta^2 - \omega^2 - 2i\Gamma\omega} \frac{1}{-(\omega + i\kappa/2)^2 + \omega_f^2}.$$

(S17)

From this equation it is clear that the linear spatiotemporal HRF contains only two new model parameters that do not occur in purely temporal models of hemodynamics, such as the balloon

model. These are the strength of viscous damping, D (which is included in the damping constant Γ) and the propagation speed v_β.

Calculation of the BOLD response

The transfer function, Eq. **S17**, can be used to derive the BOLD response to an arbitrary neural input via the following steps: (i) Fourier transform the neural activity input from **r** and t to **k** and ω.; (ii) Use the transfer function, Eq. **S17**, to find the corresponding BOLD response in Fourier space. (iii) Inverse Fourier transform this response back to coordinate space. These steps are expressed mathematically as:

$$y(\boldsymbol{r},t) = \int \frac{d^2\boldsymbol{k}}{(2\pi)^2} \int \frac{d\omega}{2\pi} \mathrm{T}_{\mathrm{yz}}(\boldsymbol{k},\omega) z(\boldsymbol{k},\omega) e^{-i(\omega t - \boldsymbol{k}\cdot\boldsymbol{r})}. \tag{S18}$$

In predicting the response to a 1-dimensional stimulus - for example an isoeccentric line in V1 - the following form for the neural activity is made,

$$z(\boldsymbol{r},t) = z(x,t), \tag{S19}$$

which represents a spatial distribution of neural activity that does not depend on (i.e., is constant with respect to) the spatial coordinate y' parallel to the stimulus line (where the prime in y' is used to disambiguate this from the BOLD signal variable). In Fourier space the neural activity is then given by,

$$z(\boldsymbol{k},\omega) = \delta(k_{y'}) z(k_x,\omega), \tag{S20}$$

where δ is the Dirac delta function and k_x and $k_{y'}$ are the spatial frequencies in the x and y' directions. Therefore the one dimensional spatiotemporal HRF to a 1D neural stimulus is given by the inverse Fourier transform (to within a constant factor),

$$y(x,t) = \int \frac{dk_x}{2\pi} \int \frac{d\omega}{2\pi} \mathrm{T}_{\mathrm{yz}}(k_x,\omega) z(k_x,\omega) e^{-i(\omega t - k_x x)}, \tag{S21}$$

where the delta function in Eq. **S20** has been used to evaluate the y' transform. Eq. **S21** yields the response to a line stimulus on the cortex, such as that evoked by an isoeccentric curve in the visual field.

To represent the experiment, the following form for z was used:

$$z(x,t) = b(t)\exp\left[-x^2/(2\sigma^2)\right], \quad \textbf{(S22)}$$

where $\sigma = 1$ mm is the spatial spread of the neural activity and $b(t)$ is the temporal evolution of the stimulus, which is chosen to match the experimental design, i.e.,

$$b(t) = \begin{cases} 1, & 0 < t \leq 8 \text{ s} \\ 0, & t > 8 \text{ s} \end{cases}. \quad \textbf{(S23)}$$

Model variables and parameters

The model contains physiological variables and parameters. Table S1 summarizes the model variables, and details the complete set of physiological parameters.

	Symbol	Nominal value/ range	units	source	notes
Model Variables					
Mass density contributed by blood in tissue	ξ	30–80	kg m^{-3}	[2]	
Fluid velocity	$\boldsymbol{v}$	0–12	mm s^{-1}	[3]	
Blood inflow	F	0.005-0.015	s^{-1}	[4]	
dHb concentration	Q	6–20	mmol m^{-3}	[4]	
Bold signal	y	-	-	-	
Neural activity	z	-	-	-	
Pore pressure	P	8–13	kPa	***See text***	
Full Model parameters					
Pressure coupling constant	c_1	$6\text{x}10^{-8}$	-	***See text***	a
Porous coupling constant	c_2	$10\text{x}(0.03)^{\beta}$	Pa $kg^{-\beta}m^{-3\beta}$	***See text***	a
Flow signal decay rate	κ	0.65	s^{-1}	[5]	b,c
Flow-dependent elimination constant	γ	0.41	s^{-2}	[5]	b
Mass density of blood	ρ_f	1062	kg m^{-3}	[6]	a
Effective viscosity of blood	D	106 – 850	kg m^{-3} s^{-1}	***See text***	a
Grubb's exponent	α	0.31	-	[5]	b
Elasticity exponent	$\beta = 1/\alpha$	3.2	-	*derived quantity*	a,c
Resting blood extraction fraction	ρ_{Hb}	0.4	-	[7]	b
Mean hemodynamic transit time	τ	1-4	s	[7]	b,c
Rate of oxygenation as per unit time	$\eta=\rho_{Hb}/\tau$	0.1-0.4	s^{-1}	*derived quantity*	b,c
Concentration of	ψ	1.9	mmol kg^{-1}	***See below***	

hemoglobin per unit mass density of blood.					
Resting blood volume fraction	V_0	0.03	-	[5]	b,c
Resting mass density contributed by blood in tissue.	$\xi_0 = \rho_f V_0$	30	kg m^{-3}	*derived quantity*	a
Magnetic field parameters at 3T and TE = 30 ms	k_1, k_2, k_3	4.2,1.7,0.41	-	[8]	b,c
Linearized Model parameters					
Flow natural frequency	$\omega_f = [(-\kappa^2+4\gamma)^{1/2}]/2$	0.55	s^{-1}	*linear result*	b,c
Blood outflow constant	$c_P = F_0/P_0$	–	s^{-1} Pa^{-1}	*derived quantity*	a
Ratio of resting oxygenated Hb to dHb	$\frac{\psi\xi_0}{Q_0} = \frac{\eta\tau+1}{\eta\tau}$	2-4	-	*derived quantity*	a
Damping constant	$\Gamma = \frac{1}{2}\left(\frac{\beta}{\tau} + \frac{D}{\rho_f}\right)$	0.1 – 1	s^{-1}	*Estimated in this study* (**see below**)	a,c
Propagation velocity	$v_\beta = \left(c_1 c_2 \beta \xi_0^{\beta-1}\right)^{1/2}$	1-20	mm s^{-1}	*Estimated in this study* (**see below**)	a,c
Spread of neural activity	σ	1	mm	[9]	a,c

Table S1: The model variables and parameters. At each row, the first column details the quantity, the second column show the symbols that describe these quantities in the model. The third column details the nominal range (if it exists in the literature), with its appropriate units in the fourth column and its source in the 5th column. The last column details notes on each variable/parameter: a do not have a direct analogue in the balloon model, b is a parameter used in previous balloon models, c are the set of independent variables needed to calculate the stHRF.

Ranges for damping and propagation velocity

The theory shows that calculation of spatiotemporal properties of the response requires two parameters in addition to those present in the balloon model, for example. A priori constraints on these parameters, the propagation velocity v_β and temporal damping rate Γ, can be made by comparisons with previous experimental work. This means that, although prior work does not give precise values for these quantities, it does yield their approximate values and prevents them from being treated as free parameters.

Firstly, as mentioned in the main text, pressure changes occur on scales of order the spacing of 0.75-4 mm between arterioles [10] in times of order hemodynamic transit time, τ=1-4 s. Hence a priori estimates of v_β are in the order 1 mm s^{-1}.

The temporal damping rate Γ has two components, the average viscous damping and the contribution from loss due to outflow, with

$$\Gamma = \frac{1}{2}\left(\frac{\beta}{\tau} + \frac{D}{\rho_f}\right). \tag{S24}$$

The first term in **S24** is from the outflow condition. Using the physiological values in **Table S1** this contribution lies in the range 0.25 – 3 s^{-1}. The second contribution involves *D*, which parameterizes blood viscosity. Previous experimental work shows that [3]:

- Average artery Red blood cell velocity (RBV) is ~ 12 mm s^{-1}
- The red blood cell velocity drop (ΔRBV) from artery to vein is around 6 mm s^{-1}
- ΔRBV from artery to capillary is around 10 mm s^{-1}

Now this viscous term occurs on average for a compartment so it will occur of the hemodynamic transit time, τ, so then the estimate for D/ρ_f is

$D/\rho_f \sim (\Delta RBV/RBV)/\tau$

Therefore this term ranges from ~0.1 – 0.8 s^{-1}, therefore the a priori estimate of Γ lies in the range

$$0.2\ s^{-1} < \Gamma < 2\ s^{-1}. \tag{S25}$$

Determination of non-linear model parameters

The concentration of hemoglobin in tissue is approximately 56 mmol m^{-3} [11], The model specifies that the concentration of total hemoglobin is $\psi\xi$. Therefore at rest,

$$\psi\xi_0 = 56\ \text{mmol m}^{-3}, \tag{S26}$$

and using the tabulated value for ξ_0, ψ is approximately 1.9 mmol kg^{-1}.

On the ranges of pore pressure, *P*: Typical values between artery and vein which range from 8 – 13 kPa [12]. At rest, the average value of 10kPa, this is inserted into the constituent equation at rest,

$$P_0 = c_2\, \xi_0^{\beta}\,, \qquad \textbf{[S27]}$$

to find that $c_2 \sim 0.4\ m^9\ Pa\ kg^{-3}$.

Regarding the pressure coupling constant c_1, the properties of the microvasculature are considered and extended to the mean field. Firstly, the pressure coupling in the microscale, C_1, is related to c_1 by $c_1 = C_1\phi$, where ϕ is the porosity. This shows that the pressure will couple to a fraction of the total tissue volume and is approximately V_0. Secondly, the momentum (Eq. **3** of the main text] is at rest,

$$C_1 \nabla P = -Dv\,, \qquad \textbf{[S28]}$$

where the velocity terms, due to the boundary inflows/outflows, present in Eq. **3** of the main text are absorbed to deal with the effective velocity *v.* This then provides estimates for the pressure coupling in the microscale C_1. To find a value for C_1, one can consider the following properties:

- The pressure drop from artery to vein is ~ 5300 Pa (40 mm Hg) [12], which is assumed to occur over the order of artery to vein spacing ~1mm [10].
- Red Blood Cell velocity, is in the order of 10 mm s-1 [3], which is attributed to the effective velocity *v*.

Using these arguments, and that $D/\rho_f \sim 1\ s^{-1}$(see above) an approximate value for C_1 is 2×10^{-6}, therefore c_1 is approximately 6×10^{-8}. Furthermore the estimates for c_1 and c_2 again imply that v_β is of order mm s^{-1}.

Polynomial fitting of the expected central response

The estimation of the centerline for each subject was made by a polynomial fit to each subject, where the fit was made by a nonlinear least squares fit (**Figure S3**). In all cases, the higher the polynomial order *n*, the lower the residual error, at a price of higher degrees of freedom. The value of *n* was chosen by optimizing the Akaike Information Criteria (AIC) [13]. The AIC penalizes models (i.e. different *n*) with a higher complexity and rewards models with lower residual error. The AIC was calculated by

$$\mathrm{AIC}(n) = 2(n+1)+N\log(\sigma_n), \quad \textbf{[S29]}$$

where n is the degree of the polynomial, N is the total number of points fitted, and σ_n is the standard deviation of the residual error of the fit for degree n.

References

1. Drysdale P, Huber JP, Robinson PA, Aquino KM (2010) Spatiotemporal BOLD hemodynamics from a poroelastic hemodynamic model. J Theor Biol 265: 524-534.

2. Rostrup E (2005) The relationship between cerebral blood flow and volume in humans. Neuroimage 24: 1-11.

3. Lipowsky HH (2005) Microvascular rheology and hemodynamics. Microcirculation 12: 5–15.

4. Toronov V, Walker S, Gupta R, Choi JH, Gratton E, et al. (2003) The roles of changes in deoxyhemoglobin concentration and regional cerebral blood volume in the fMRI BOLD signal. Neuroimage 19: 1521-31.

5. Friston KJ, Harrison L, Penny W (2003) Dynamic causal modeling. Neuroimage 19: 1273-1302.

6. Trudnowski RJ, Rico RC (1974) Specific gravity of blood and plasma at 4 and 37 degrees C. Clin Chem 20: 615-6.

7. Buxton RB, Uludag˘ KM, Dubowitz DJ, Liu TT (2004) Modeling the hemodynamic response to brain activation. NeuroImage 23: S220 – S233.

8. Huppert TJ, Hoge RD, Diamond SG, Franceschini MA, Boas DA (2006) A temporal comparison of BOLD, ASL, and NIRS hemodynamic responses to motor stimuli in adult humans. NeuroImage 29: 368 – 382.

9. Blasdel G, Campbell D (2001) Functional Retinotopy of Monkey Visual Cortex. J Neurosci 21: 8286-8301.

10. Duvernoy H, Delon S, Vannson J (1981) Cortical blood vessels of the human brain. Brain Res Bull 7: 519–79.

11. Zheng Y, Johnston D, Berwick J, Chen D, Billings S, et al. (2005) A three-compartment model of the hemodynamic response and oxygen delivery to brain. Neuroimage 28: 925-939.

12. Buxton RB (2009) Introduction to Functional Magnetic Resonance Imagaing: Principles and Techniques. New York: Cambridge University Press.

13. Akaike H (1974) A new look at the statistical model identification. IEEE Trans Automat Contr 19: 716–723.
